# Evaluating the Binding Potential and Stability of Drug-like Compounds with the Monkeypox Virus VP39 Protein Using Molecular Dynamics Simulations and Free Energy Analysis

**DOI:** 10.3390/ph17121617

**Published:** 2024-11-30

**Authors:** Ahmed M. Hassan, Hattan S. Gattan, Arwa A. Faizo, Mohammed H. Alruhaili, Azzah S. Alharbi, Leena H. Bajrai, Ibrahim A. AL-Zahrani, Vivek Dhar Dwivedi, Esam I. Azhar

**Affiliations:** 1 Special Infectious Agents Unit—BSL3, King Fahd Medical Research Center, King Abdulaziz University, Jeddah 21362, Saudi Arabia; hmsahmed@kau.edu.sa (A.M.H.); hsqattan@kau.edu.sa (H.S.G.); asalharbi3@kau.edu.sa (A.S.A.); iaalzahrani1@kau.edu.sa (I.A.A.-Z.); 2 Medical Laboratory Sciences Department, Faculty of Applied Medical Sciences, King Abdulaziz University, Jeddah 21362, Saudi Arabia; 3 Department of Clinical Medical Microbiology and Immunology, Faculty of Medicine, King Abdulaziz University, Jeddah 21362, Saudi Arabia; 4 Department of Biochemistry, Faculty of Sciences, King Abdulaziz University, Jeddah 21362, Saudi Arabia; 5 Center for Global Health Research, Saveetha Institute of Medical and Technical Sciences, Saveetha Medical College and Hospitals, Saveetha University, Chennai 602105, India; 6 Bioinformatics Research Division, Quanta Calculus, Greater Noida 201310, India

**Keywords:** monkeypox, VP39 protein, computational drug discovery, molecular dynamics simulations, antiviral agents

## Abstract

**Background/Objectives:** Monkeypox is a re-emerging viral disease with features of infectiously transmitted zoonoses. It is now considered a public health priority because of its rising incidence and transmission from person to person. Monkeypox virus (MPXV) VP39 protein is identified as an essential protein for replication of the virus, and therefore, it is a potential target for antiviral drugs. **Methods:** This work analyzes the binding affinities and the differential conformational stability of three target compounds and one control compound with the VP39 protein through multiple computational methods. **Results:** The re-docking analysis revealed that the compounds had high binding affinities towards the target protein; among these compounds, compounds **1** and **2** showed the highest binding energies in the virtual screening, and thus, these were considered as the most active inhibitor candidates. Intermolecular interaction analysis revealed distinct binding mechanisms. While compound **1** had very strong hydrogen bonds and hydrophobic interactions, compound **2** had numerous water-mediated interactions, and compound **3** had only ionic and hydrophobic contacts. In molecular dynamic simulations, compounds **1** and **2** showed that the protein–ligand complexes had a stable conformation, with protein RMSD values around 2 Å for both compounds. In contrast, compound **3** was slightly flexible, and the control compound was more flexible. MM/GBSA analysis again supported these results, which gave the binding free energies that were also supportive for these compounds. **Conclusions:** Notably, all the selected compounds, especially compounds **1** and **2**, demonstrate high binding affinity. Therefore, these compounds can be further tested as antiviral agents against monkeypox treatment.

## 1. Introduction

Monkeypox is a newly identified zoonotic disease closely related to MPXV in the Orthopoxvirus family [1,2]. The virus was first isolated in 1958 in laboratory monkeys. It was discovered to cause human disease, resulting in infrequent occurrences and sporadically documented outbreaks in the academic literature, especially in the Central and West African Regions [3,4]. Nonetheless, geographic occurrence and the spreading of monkeypox have increased in the recent past, thus posing a threat to humanity by causing a pandemic. The World Health Organization (WHO) has observed rising disease incidence in other continents, especially in areas where the virus was once unknown [5]. This transition emphasizes the requirement to improve the identification and monitoring of infection emergence and pathogen circulation, prepare for the subsequent waves of viral spread, and design appropriate antiviral treatment strategies.

There are many natural reservoirs of monkeypox, the most common of which is rodents; intermediate hosts include squirrels, gerbils, porcupines, etc. [6]. The infection spreads through direct contact with infected animals or their products, or indirect contact with infected animals or their products, respectively, while the latter can be through contact with respiratory droplets, bodily fluids, or contaminated articles. Symptoms of monkeypox in humans are similar to those of smallpox. However, monkeypox is considered less lethal and presents with fever, fatigue, muscle weakness, but most obviously, a rash that develops through phases [7]. However, dangerous effects are possible, especially for immunocompromised patients, children, and pregnant women, so the question of the pathogenesis of the virus and the search for potential therapeutic targets for the virus remains relevant. Monkeypox virus has double-stranded DNA, and its genome makes it approximately 197.2 kilobases in size with over 181 proteins coded [8,9]. As with other Ortho poxviruses, the genomic structure of MPXV is similar to variola, which causes smallpox. This disease is eradicated [10]. The organization of the MPXV genome is seen with a central conserved core that contains genes that are indispensable for virus replication. At the two terminal extremities, there are genes that determine the host range and pathogenicity. Additionally the viral enzymes that play the roles in DNA synthesis, transcription, and modification of viral proteins can be considered as promising targets for treatments [11]. An enzyme known as VP39 viral methyltransferase is involved in the capping and methylation steps that are critical in protein synthesis and immune evasion in that it prevents degradation of viral mRNA by the host cell’s enzymes [12]. VP39 was identified as an S-adenosylmethionine (SAM)-dependent methyltransferase that methylates the 2-O position of the viral mRNA 5′ cap, facilitating the protection and translation of the viral RNA. These findings suggest that VP39 is important in viral replication, thus constituting an attractive target for antiviral therapeutics.

Several studies were conducted on small molecules that could inhibit VP39, a valuable target for monkeypox [13]. One of the identified inhibitors, TO427, has shown some promising therapeutic activity and thus holds potential for future advancement as it can bind to the active site of VP39 [14]. Even though TO427 could be proven to effectively inhibit VP39 of the monkeypox virus, existing monkeypox antiviral treatment still needs to be made available [15]. Until now, there have been no specific treatments for MPXV infection, and the main approaches to management have been supportive of the use of smallpox vaccines, which offer some level of protection against MPXV. Specific antiviral drugs, such as TO427, should be produced to enhance patient treatment outcomes, especially given the emerging global spread of the virus and the potential for more severe clinical presentations. These data highlight the identification of VP39 inhibitors, as a potential strategy for designing target-specific approaches for monkeypox treatment [16]. Diverse Lib is a powerful tool to enrich the discovery and development of novel therapeutics in the chemical compound database. The Diverse Lib library comprises small bioactive molecules, such as different enzymes and receptors’ inhibitors, which can be used to understand some significant biological processes. The database is applicable to virtual screening and structure–activity relationship analysis, which allows us to find and optimize leads quickly and easily. Thus, the current study provides information on the potential inhibitors of the monkeypox virus, particularly targeting the VP39 protein, using a compounds database known as the Diverse Lib database based on a computational approach, which can be further exploited for the development of efficient antiviral drugs.

## 2. Result

### 2.1. Structural-Based Virtual Screening

Virtual screening is an important computational approach used to discover active compounds that possess likely binding similarity to a specific receptor attributable to biological activities from large databases of compound libraries. In the current study, the MTiOpenScreen web server virtual screening as a method to screen compounds from the Diverse Lib library was utilized [17]. The number of compounds obtained from this search was 1500 after identifying the best hits with binding energy from the screening phase. Out of these 1500, a critical evaluation was performed, and all of these compounds showed binding energies that varied between −10.8 kcal/mol and −8.3 kcal/mol, as is presented in the Supplementary Table S1. From these, four compounds, compound **1** (17444176), compound **2** (17450998), and compound **3** (24392109), were chosen for further analysis.

### 2.2. Redocking and Intermolecular Analysis and ADME Analysis

**Figure 1:**
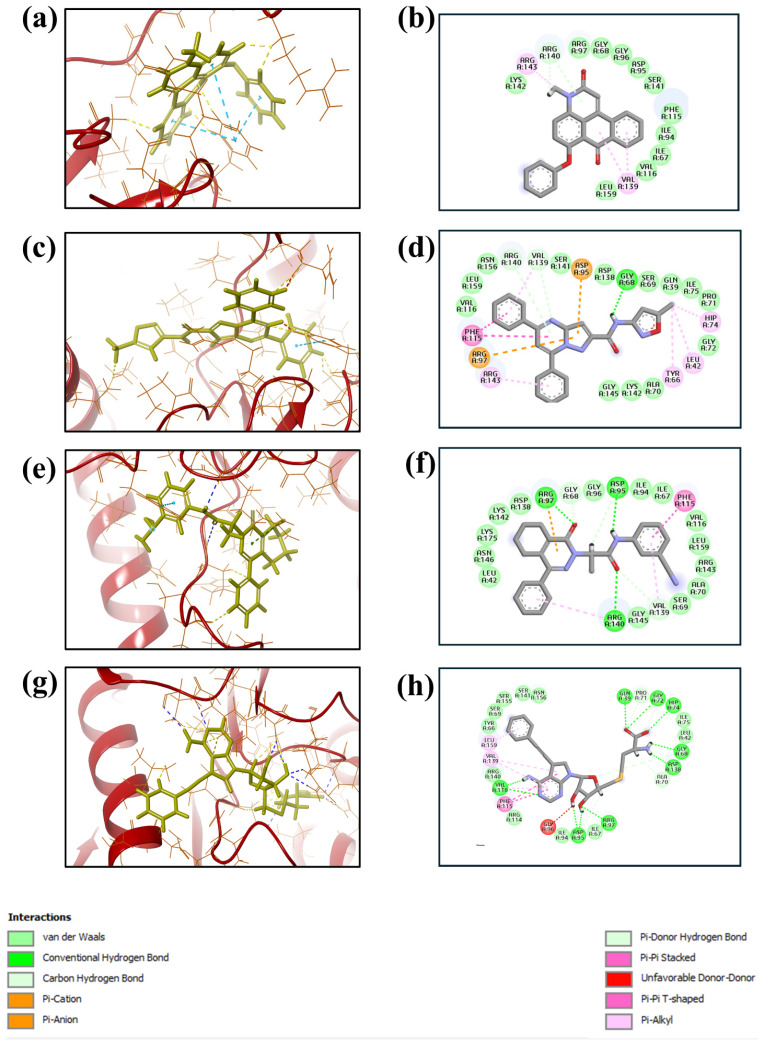
Three-dimensional and two-dimensional structure analysis of four selected compounds in the docked pocket of selected compounds, i.e., (**a**,**b**) compound **1**, (**c**,**d**) compound **2**, (**e**,**f**) compound **3**, and (**g**,**h**) the control. The red color in ligand represents oxygen, while the blue color represents nitrogen, and the yellow color represents Sulfur.

Based on the docking scores obtained from the virtual screening, the three most negative binding energy compounds were selected for the redocking process in addition to the control molecule to form a stable complex with the target protein [18]. The center of the docking grid was used as (X = −22.34, Y = −18.74, Z = 45.75), and the redocking grid was extended 20 Å along the X, Y, and Z axis. This redocking method validates virtual screening compounds with significant binding energy. This redocking exercise identified three compounds with significant binding energies: −10.8 kcal/mol for compound **1**, −11.0 kcal/mol for compound **2**, −8.6 kcal/mol for compound **3**, and −10.2 kcal/mol for control compound. The redocking results confirmed these compounds’ strong binding affinity to the protein and proved their suitability for further study. This method confirmed our initial criteria for selecting compounds through virtual screening and emphasized that the chosen compounds are good candidates for further experiments, as shown in Figure 1. The 3D structures were visualized using PyMOL [19]. The 2D structures were created with the visualization tool available in Discovery Studio [20]. The compounds’ interaction with the target protein is depicted in Table 1 below.

While the ADME analysis indicates that all three compounds possess favorable pharmacokinetic properties, showing potential as drug candidates with distinct strengths performed by the swissADME [21]. Compound 1 demonstrates excellent oral bioavailability, high membrane permeability, and moderate plasma protein binding, suggesting efficient absorption and balanced distribution with minimal CYP450 interactions, which enhances its metabolic stability and potentially reduces dosing frequency. Compound **2** has moderate oral bioavailability and lower protein binding, which allows broad tissue distribution. Although it undergoes significant CYP3A4 metabolism, this feature could be managed in dosing strategies, making it suitable for targeted applications. Compound **3** exhibits highly favorable properties, including strong oral bioavailability, excellent solubility, high permeability, and balanced excretion through renal and biliary pathways, minimizing the risk of accumulation. Its high plasma protein binding and minimal CYP interactions contribute to sustained activity in the bloodstream. Overall, compounds **1** and **3** show exceptional promise for oral delivery, while compound **2** may benefit from optimization for broader therapeutic potential.

### 2.3. Specificity Analysis

To confirm the specificity of compounds **1** and **2**, we extended docking studies to include two additional proteins: A42R Profilin-like Protein from Monkeypox virus (PDB ID: 4QWO) [22] and Human B-cell lymphoma-2 (Bcl-2) protein (PDB ID: 8HTS) [23]. These targets were selected based on their biological relevance.

The A42R Profilin-like Protein, a viral protein distinct from VP39, regulates cytoskeletal dynamics critical for viral replication and host–pathogen interactions [22]. It serves as a suitable viral off-target candidate to test whether the selected compounds interact with unrelated Monkeypox virus proteins. In contrast, the Bcl-2 protein, a human host protein involved in apoptosis regulation, is often exploited by viruses for immune evasion and viral persistence [24,25,26]. Evaluating the binding of compounds **1** and **2** to Bcl-2 ensures their specificity for viral targets while minimizing potential off-target effects on host proteins.

The docking results demonstrated the considerably weaker binding affinities of compounds **1** and **2** with these off-target proteins compared to VP39. The docking energies for A42R were −6.9 kcal/mol and −7.1 kcal/mol for compounds **1** and **2**, respectively, while for Bcl-2, the values were −8.3 kcal/mol and −8.1 kcal/mol. These energies are significantly less favorable compared to their binding affinities with VP39 (−10.8 to −11.0 kcal/mol).

Further analysis of intermolecular interactions corroborated these findings (Supplementary Figures S1 and S2). For A42R, compounds **1** and **2** formed limited hydrogen bonds and van der Waals interactions, indicating a lack of strong binding (Supplementary Table S2). Similarly, for Bcl-2, the interactions were sparse, and key residues required for high-affinity binding were not engaged (Supplementary Table S3).

These results confirm the high specificity of compounds **1** and **2** for VP39, as their interactions with unrelated viral and host proteins were significantly weaker. This analysis highlights the potential of these compounds as targeted antiviral agents with minimal risk of off-target effects on other proteins.

### 2.4. Dynamical Analysis

To analyze the dynamic stability and the interaction pattern of the three selected compounds in complex with VP39 protein, MD simulations were performed for 1000 ns. These compounds were chosen due to their high negative score; the molecular dynamics simulation was performed using Desmond-Maestro 2020-4, an academic-free Desmond software. We have performed the simulation of each complex in triplicates to avoid false positive conclusions in molecular simulation and calculated the standard deviation and standard error [27].

#### 2.4.1. RMSD Analysis

**Figure 2:**
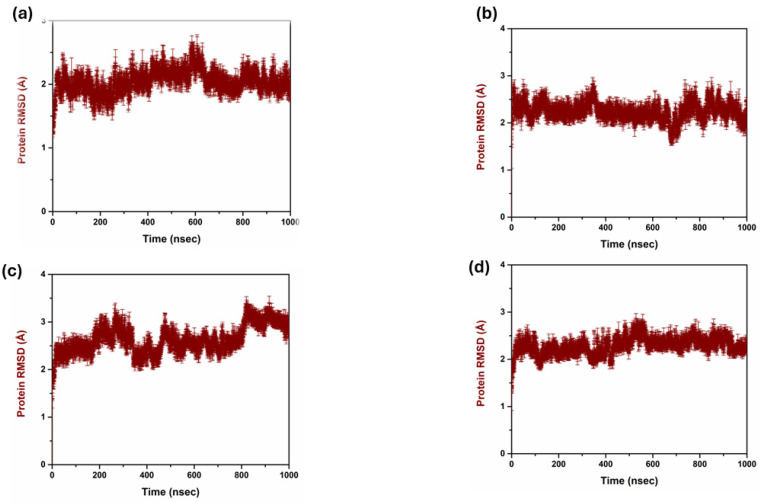
Protein RMSD triplicate in complex with three compounds and one control. (**a**) Compound **1**, (**b**) Compound **2**, (**c**) Compound **3**, and (**d**) Control.

**Figure 3:**
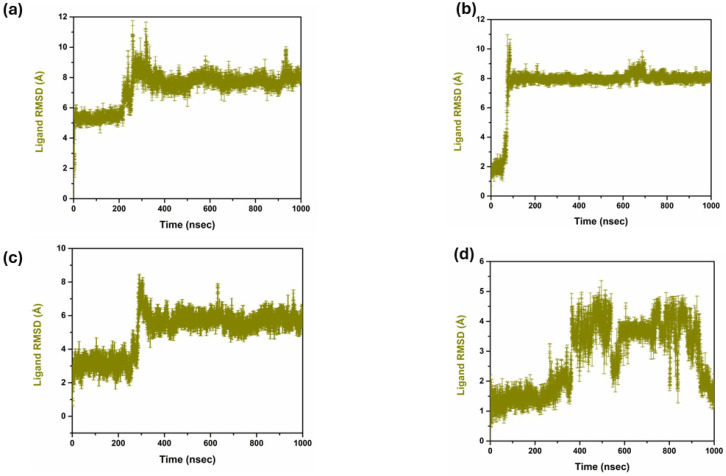
Ligand RMSD triplicate in complex with three compounds and one control. (**a**) Compound **1**, (**b**) Compound **2**, (**c**) Compound **3**, and (**d**) Control.

The analysis of the RMSD of the compounds helped to compare their structural stability and dynamic activity. Thus, the RMSD has a critical function in the drug discovery process as the application measures the stability and conformational changes of the protein–ligand complexes throughout the simulations. RMSD values are calculated and defined as the amount by which the average distances between atoms deviate from a reference structure, generally the starting structure, to evaluate the structural stability over time. Lower RMSD values are preferred as they indicate good structural stability and conformational changes are minimal, which are desirable characteristics for leads; hence, a good binder [28] is illustrated in Figure 2. The triplicate RMSD value of 1 to 2.5 was observed in the complex 1 with mean value of 2.040 and standard deviation of 0.181. While in complex 2 the protein RMSD value of 2 to 2.5 Å was observed with a mean value of 2.245 and a standard deviation of 0.193. However, in complex 3, a protein RMSD value of 2.5 to 3 Å was observed with a mean value of 2.628 and a standard deviation of 0.296. On other hand, the control complex exhibited a protein RMSD value of range between 2 and 2.5 Å and a mean value of 2.290 and a standard deviation of 0.180. Figure 3 reflects the triplicate ligand RMSD values. Complex 1 exhibited the ligand RMSD value of 7 to 8 Å, with a mean value of 7.27 and a standard deviation of 1.16. Complex 2 exhibited a ligand RMSD value of 8 to 9 Å with a mean value of 7.59 and a mean value of 7.59 and standard deviation of 1.54. On other hand, complex 3 exhibited an overall RMSD value of 5 to 7 Å. The control complex exhibited a ligand RMSD value of 1 to 2 Å until 400 ns, and from 400 to 820 ns it exhibited a ligand RMSD value of 1 to 5 Å. From 820 ns to 1000 ns, the ligand RMSD value was 1 to 2 Å with a mean value of 2.78 and a standard deviation of 1.14.

#### 2.4.2. Protein RMSF Analysis

**Figure 4:**
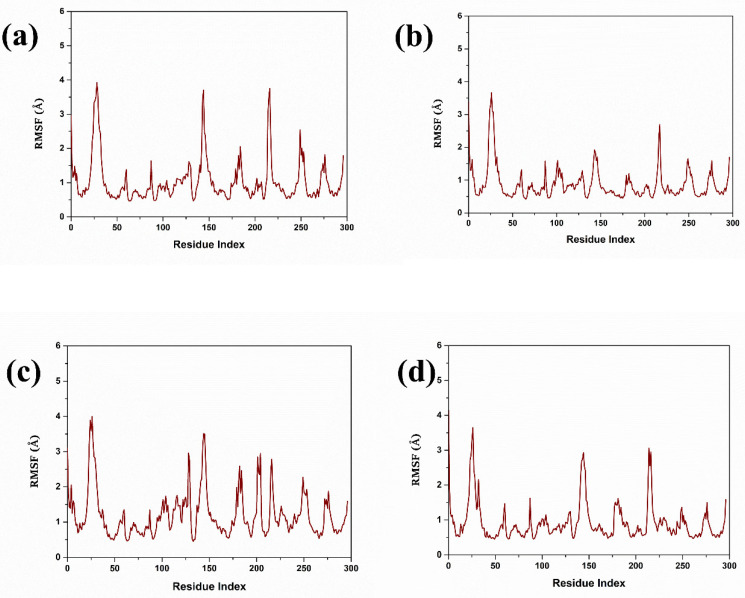
Protein RMSF of top selected compound in complex with control complex. (**a**) Compound **1**, (**b**) Compound **2**, (**c**) Compound **3**, and (**d**) Control.

The RMSF values are of great importance in the drug discovery process because they provide important data for the motion and stability of the protein structures if they interact with the different compounds, as explained in Figure 4. In most cases, lower RMSF values correspond to effective binding interactions and higher stability, indicating the protein’s stability [29]. Comparing the RMSF analysis of three targeted compounds and the control compound, **1**, **2**, **3** showed the amount of flexibility and the conformational change in protein residue in the simulation. In the case of compound **1**, the RMSF is demonstrated to increase around regions 50, 100, 150, 200, and 250, and varies up to 4 Å. This suggests that these areas of the protein show higher levels of conformational freedom in the protein, which could be attributed to conformational freedom from looking at the loops or parts of the surface area on the protein that can move more freely. However, in the case of compound **2**, the RMSF profile is almost similar to compound **1** with a slightly lower maxima fluctuation up to 4 Å. This indicates that compound **2** may constrain these flexible regions, decrease their total motility, and yet enable them to be flexible enough to allow the protein to function. On the other hand, compound **3** revealed an RMSF profile with the same patterns, with the flexibility ranging up to 4 Å, giving the same pattern of flexibility as compounds **1** and **2**. There is a marginal increase in flexibility near these two peaks, specifically between them, and this flexibility may suggest that compound **3** allows more significant movement and change within the protein structure while maintaining a rigid overall structure. In the case of the control compound, the similar peaks at nearly similar positions and similar in magnitude as observed in the earlier case with maximum fluctuations reaching the RMSF value of 4 Å. This seems to agree with the fact that the observed flexible regions are natural and the motion of the protein and are not affected by the compounds. According to the RMSF analysis, compounds **1**, **2**, **3**, and the control compounds keep the protein fold intact and possess intrinsic flexibility. The effect of each compound seems to be distinct from all the others. Still, the overall map of residue flexibility remains similar across all conditions, which indicates that protein structure and dynamics have similar characteristics.

#### 2.4.3. Ligand RMSF Analysis

**Figure 5:**
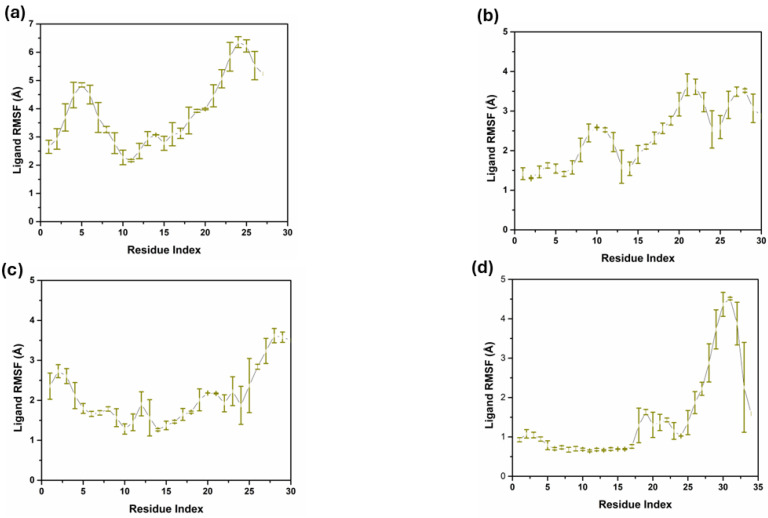
Ligand RMSF triplicate in complex with three compounds and one control. (**a**) Compound **1**, (**b**) Compound **2**, (**c**) Compound **3**, and (**d**) Control.

The RMSF analysis of the ligand atoms of compounds **1**, **2**, **3**, and the control shows that these compounds’ flexibility and dynamic behavior are slightly or significantly different when they bind to their target proteins, as shown in Figure 5. Compound **1**, the RMSF values fluctuated less than 2 Å for most ligand atoms, with a spike towards the latter ones, increasing up to 4 Å. This implies that the ligand does not undergo a major change in its conformation while having moderate flexibility and this can entail the positivity of the binding interaction with the protein. Compound **2** also did not exhibit any pattern concerning its RMSF values, which vary from about 0.3 Å to almost 4 Å, starting from the start to the middle part of the alkyl chain at atom indices 25 to 30. According to this pattern, the terminal part of the ligand is more mobile, which may be attributed to a weak interaction with the protein or solvation of the ligand, while other sections are moderately ordered. The RMSF for compound **3** is predominantly similar to compound **2**, with RMSF ranging from 1 to 2 Å, thus suggesting that the compound maintains a relatively rigid conformation for its binding site and is not very dynamic, albeit for slightly increased flexibility in the middle of the ligand. There is a difference in the RMSF profile of the control compound, with one of the prominent peaks touching nearly 4 Å at atom indices, 30 to 32, which indicates that this part is quite flexible. The remaining residues may be related to less stable or less long-lasting binding to the protein, and the other part of the ligand has low RMSF values, comparable to the other compounds. This study has shown that all ligands have a certain level of flexibility. Still, the degree and region of flexibility differ among ligands, and this has to do with the differences in binding modes and protein–ligand interactions.

#### 2.4.4. Protein–Ligand Profiling

**Figure 6:**
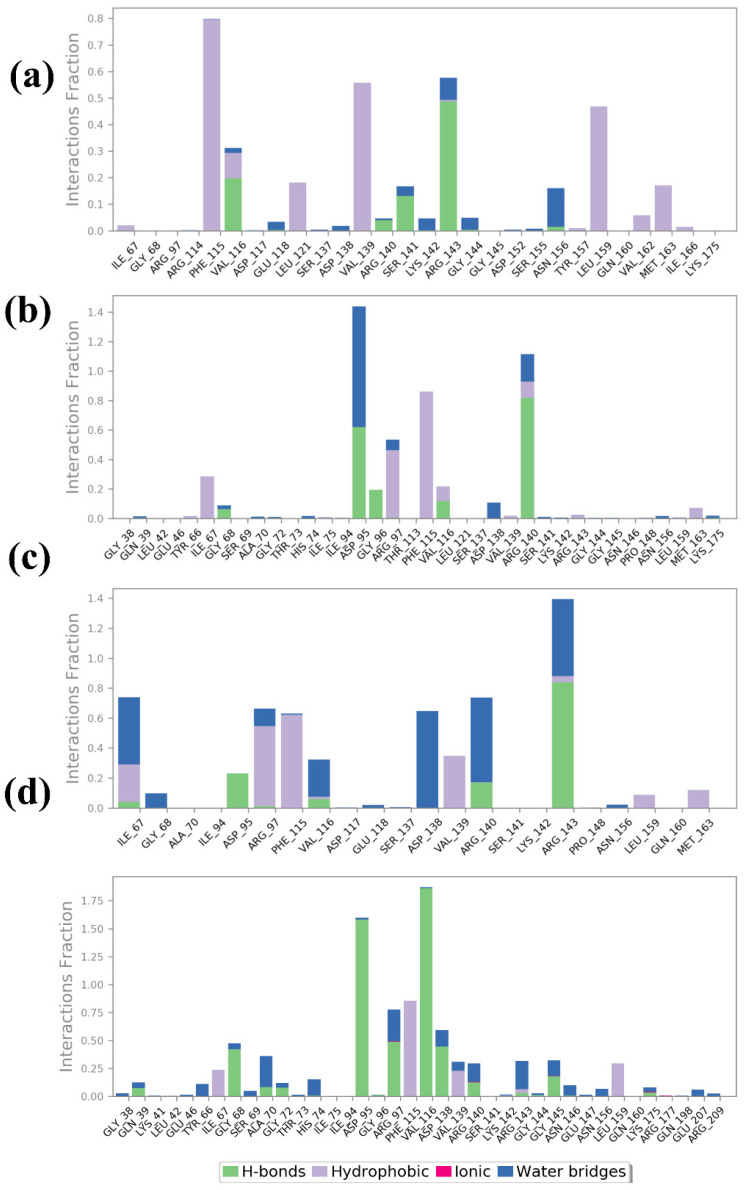
Protein–ligand profiling of selected compounds in the docked pocket of selected protein, i.e., (**a**) compound **1**, (**b**) compound **2**, (**c**) compound **3** and, (**d**) the control.

The protein–ligand interaction profiles for compounds **1**, **2**, **3**, and the control provide detailed information about the nature and strength of the interactions that stabilize each ligand within the protein binding site. These interactions are categorized into hydrogen bonds, hydrophobic interactions, ionic interactions, and water bridges, which are crucial for maintaining the binding affinity and specificity of the ligands by the Desmond software, as shown in Figure 6. Compound **1**’s interaction profile indicates a significant contribution from H-bonds and hydrophobic interactions. Notably, strong hydrogen bonds are formed with residues Val116, Ser141, and Arg143, which show high interaction fractions. This suggests that these residues are vital in anchoring the ligand through polar interactions. Hydrophobic interactions are also prominent, particularly with residues Phe115, Val139, and Leu159, indicating that nonpolar contacts contribute significantly to stabilizing the ligand within the binding pocket. Water bridges were also observed to contribute moderately, indicating solvent-mediated interactions that further stabilize the binding. Compound **2** exhibits a different interaction pattern, with a notable increase in water bridge interactions, particularly involving residues Asp95 and Glu147. The blue bars indicate that these residues are involved in solvent-mediated contacts crucial for stabilizing the ligand. There is also a significant presence of hydrophobic interactions with residues Val93, suggesting that nonpolar interactions are important for ligand binding. Although present, hydrogen bonds with residues Asp95 and Arg140 are less frequent than in compound **1**, indicating a different balance of interaction types that stabilize this ligand within the protein. In compound **3**, water bridges and hydrogen bonds dominate the interaction profile, indicating a strong reliance on polar interactions for ligand stabilization. Key residues involved in hydrogen bonding include Asp95, Arg140, and Arg143, which form multiple polar contacts. The water bridges are also significant, particularly involving residues Gly68, Ala70, Asp138, Arg97, and Arg143, suggesting that solvent-mediated interactions play a major role in stabilizing the ligand. Hydrophobic interactions are moderate, with some contribution from residues Ile67, Phe115, and Val139, indicating a mixed mode of interaction where both polar and nonpolar contacts are essential. The control compound showed a more balanced interaction profile, with substantial contributions from all four types of interactions. Hydrophobic interactions are notably strong with residues Ile67, Phe115, Val139, and leu159, suggesting that nonpolar contacts are a major stabilizing force for the control ligand. Hydrogen bonds are also significant, particularly with residues Gly68, Asp95, Val116, and Asp138, which indicate strong polar interactions. Additionally, there are notable ionic interactions with residues Arg140, highlighting the importance of electrostatic attractions. Water bridges also contribute significantly, particularly involving residues Arg97, Arg143, and Gly145, indicating a robust network of solvent-mediated contacts that further stabilize the ligand.

**Figure 7:**
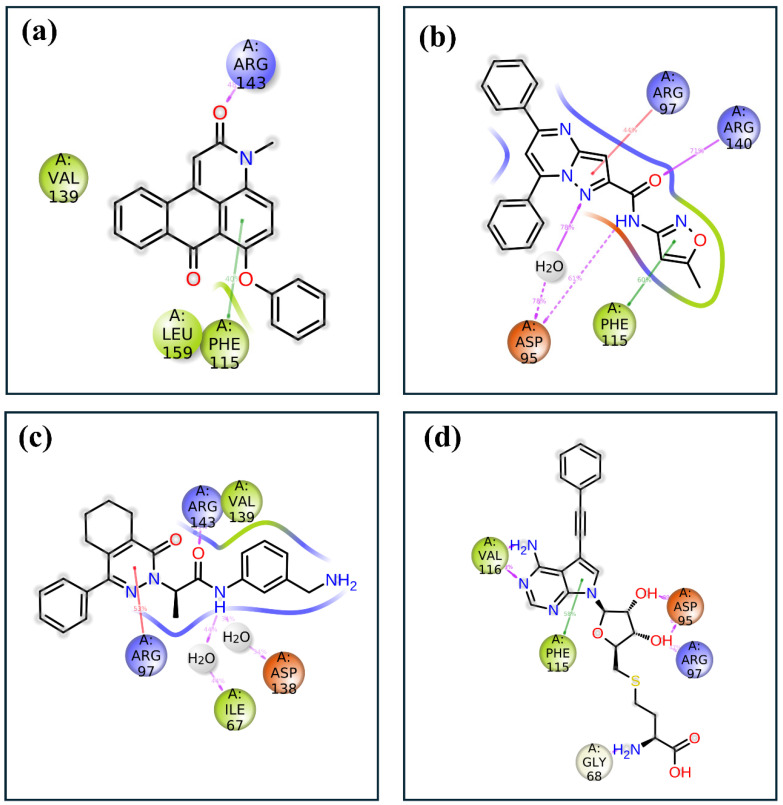
Two-dimensional protein–ligand interaction analysis of selected compounds in the docked pocket of target protein, i.e., (**a**) compound **1**, (**b**) compound **2**, (**c**) compound **3** and, (**d**) the control.

The 2D interaction diagrams of the protein–ligand complexes for the four compounds provide a detailed view of the key residues involved in binding and their respective interaction frequencies, highlighting the nature and strength of these interactions, as shown in Figure 7. In compound **1**, the ligand is predominantly stabilized by hydrophobic interactions with residues such as Phe115, which shows the highest interaction frequency at approximately 40%. Arg143 also contributes significantly, forming hydrogen bonds with about 60% frequency, underscoring its importance in polar interaction networks. In compound **2**, the ligand binding is characterized by a diverse range of interactions, including hydrophobic contacts with Phe115 at around 60% frequency and significant hydrogen bonding and ionic interactions involving Asp95 and Arg97, contributing approximately 50% and 40%, respectively. Additionally, Arg140 participates in stabilizing the ligand through hydrogen bonds, adding about 30% to the overall interaction profile. A combination of hydrophobic, hydrogen bonding, and ionic interactions supports compound **3**’s binding. Val139 and Arg143 play crucial roles, with 60% and 55% interaction frequencies, respectively. Asp138 forms an ionic interaction with approximately 50% frequency, further stabilizing the complex, while Ile67 and Arg97 contribute additional hydrophobic and water-mediated interactions. The control compound exhibits a more varied interaction profile, with strong ionic interactions from Asp95 and Asp97, contributing approximately 70% and 60% to the binding interactions. Hydrophobic contacts are also significant, with Phe115 and Val116 showing frequencies of around 50% and 40%. Gly68 provides further stabilization with about 30% interaction frequency, reflecting a robust network of both ionic and hydrophobic interactions.

#### 2.4.5. SASA Analysis

**Figure 8:**
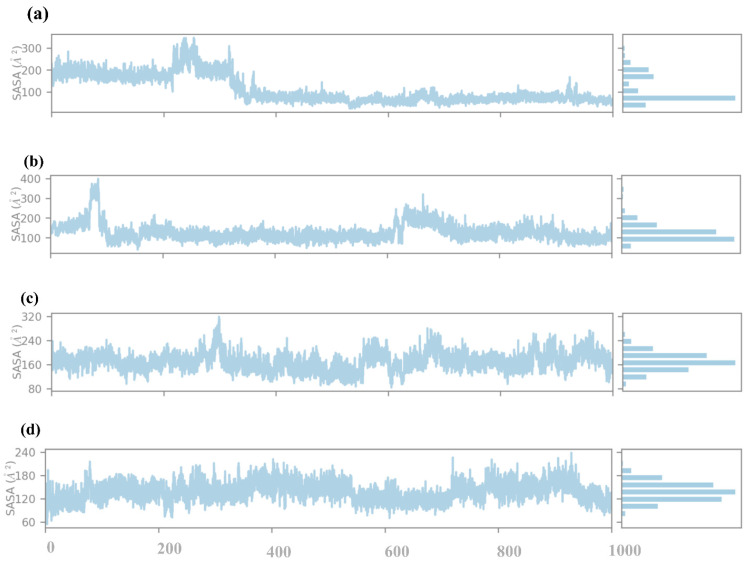
SASA analysis of selected compounds in the docked pocket of target protein, i.e., (**a**) compound **1**, (**b**) compound **2**, (**c**) compound **3** and, (**d**) the control.

The SASA analysis of compounds **1**, **2**, **3**, and the control to obtain information on how far the ligand is exposed to the solvent during the simulation. SASA is an essential parameter that determines the percentage of burial of the ligands in a protein binding pocket, i.e., the extent of ligand exposure to solvent molecules, as shown in Figure 8. Compound **1**, the SASA profile, reveals the first peak in the early region with the SASA values in the vicinity of 300 Å^2^. This means that in the initial state of the simulation, the ligand has a higher probability of coming into contact with the solvent, implying that it could be partially outside or weakly trapped within the protein binding pocket. With the increase in the simulation time, SASA values gradually decline, and at the end of the simulation, SASA values are around 100 Å^2^. This observation of the gradual reduction in solvent exposure over time indicates that the ligand is positioned deeper within the binding pocket, thus implying tighter binding to the protein. The decreasing pattern of SASA observed in the later part of the simulation shows that the ligand is likely to have a stable conformation with the least exposure to solvent. Compound **2** has distinctly different SASA behavior, with its profile showing a dramatic drop during the initial stage of the simulation from about 400 Å^2^ down to close to 200 Å^2^. This rapid decrease further implies that the ligand experiences a large change in orientation during simulation, thereby entombing itself within the protein structure. SASA values after this adjustment remain constant and oscillate around 200 Å^2^, thus suggesting a relatively stable interaction between the ligand and the protein with moderately open surface to solvent. The downward trend of the SASA indicates that compound **2** may first bind in a more exposed conformation than the one observed before stabilizing into the conformation observed in the complex. In compound **3**, the SASA profile is relatively stable throughout the simulation, with values fluctuating around 320 Å^2^. This consistent SASA suggests that the ligand maintains a steady degree of solvent exposure, indicating a binding mode where the ligand is neither fully buried nor excessively exposed. The moderate fluctuations around this value suggest that the ligand might experience some conformational flexibility within the binding pocket. Still, it largely retains a consistent level of exposure to the solvent. This profile indicates that compound **3** maintains a stable albeit partially solvent-accessible binding conformation throughout the simulation. The control compound presents the SASA profile with relatively low values, consistently fluctuating around 180 Å^2^. This suggests that the control ligand is mainly buried within the protein binding pocket, with limited solvent exposure. The low and stable SASA values indicate a strong and stable binding interaction, with the ligand being well-encapsulated within the protein environment. This burial level suggests that the control ligand forms stable interactions that shield it from the solvent, likely contributing to its stability and binding affinity.

#### 2.4.6. RG Analysis

**Figure 9:**
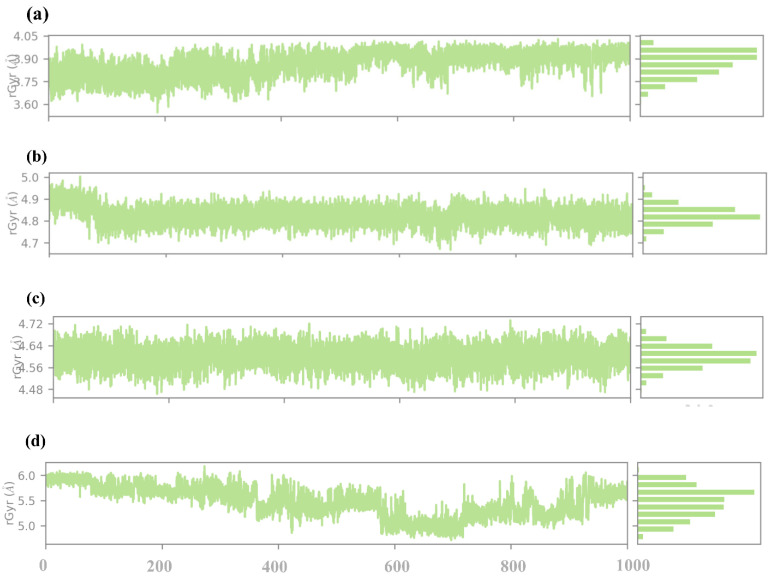
RG analysis of selected compounds in the docked pocket of the target protein, i.e., (**a**) compound **1**, (**b**) compound **2**, (**c**) compound **3** and, (**d**) the control.

The radius of gyration (RG) analysis is considered an informative measure of the protein–ligand complex’s overall compactness and conformational stability during the simulation. The RG is defined as the root mean square distance of the atoms from their centroid; hence, it can be used to measure the folding and unfolding events or of the structural changes that occur in the protein on binding the ligand, as illustrated in Figure 9. In compound **1**, the RG values fluctuate around an average comparative value of about 3.8 Å during the simulation. Here, the comparatively small value of the RG index also hints at a tight and well-packed structure of the protein–ligand complex. The small variations suggested that there must be some capacity for the protein structure to change. Compound **2** has a higher RG that is on average 4. 8 Å, which is more significant than in compound **1**. The Rg values, however, manifest higher fluctuation, going up to 4. 7 Å to 5. 0 Å, indicating that all the protein–ligand complex moieties are more flexible than the native state. This higher Rg value implies or relates to the binding of compound **2**, which may lead to a slight increase in the protein volume. Certain flexible protein structures might be released due to less stable or fewer interactions between the ligand and the protein. In compound **3**, the Rg values are not very much affected and seem to fluctuate around 4.6 Å throughout the simulation. These observations lead to the conclusion that the structure of the protein–ligand complex is rather stable in terms of a moderate level of compactness. The RG values are smaller than those determined for compound **2**, and therefore, it might be stated that the protein conformation is more compact, though the values are somewhat higher than that for compound **1**. This can be explained by the ligand binding leading to some changes in the protein conformation while remaining stable. The slight fluctuation movements that characterize the Rg indicate that the ligand enables flexibility while preventing a large increase in the protein’s size. As for the control compound, the Rg values were the highest and constantly varied around 5.5 Å. This suggests that the protein–ligand complex occupies a relatively larger space and, hence, is less compact than the rest of the compounds. The comparatively higher Rg values mean that the control ligand may cause more dramatic conformational changes, whether the binding site is more accessible, or the protein is more flexible. Given the fact that the Rg for the control is of a higher value and is varying, it is possible that the nature of ligand–protein binding interactions may not be too strong.

### 2.5. Free Binding Energy Analysis

The MM/GBSA calculations do not only afford the estimates for the binding free energies but also illustrate components of energy participating in the interactions between the target protein and the three champion compounds, namely, 17444176, 17450998, 24392109, and the control compound. The equation ΔGbind is the binding free energy of a ligand meaning that the lower the ΔGbind is, the more positive the interaction with the protein.

Compound **1** has the best ΔGbind score of −87.84 kcal/mol and the control compound has slightly lower ΔGbind score of −76.46 kcal/mol as compared to the remaining compounds. The ΔGbind value of compound **2** is −69.06 ± 4.84 kcal/mol suggesting moderate binding interaction with the protein. However, compound **3** shows the least binding affinity to the receptor among all the compounds used in the study with ΔGbind of −59.55 ± 3.99 Kcal/mol as indicated in Table 2.

The free binding energies for replica 2 and replica 3 exhibit comparable values reinforcing the consistency and reliability of these findings as detailed in Supplementary Tables S4 and S5.

### 2.6. Negative Control Analysis

To validate our findings, we selected the last compound (PubChem CID: 22406971) from the virtual screening results, having a docking energy of −8.3 kcal/mol. This compound exhibited poor binding affinity, and molecular interaction compared to the selected compounds and the control molecule (Supplementary Figure S3). MD simulations were conducted using the methodology described for the three selected compounds and the control molecule. The simulation results demonstrated significantly weaker binding and less stability for the negative control. Further analysis, including RMSD, RMSF, SASA, and radius of gyration (Rg), consistently supported the negative control’s weaker binding and lower stability (Supplementary Figures S4–S9). For instance, MM/GBSA binding energy analysis revealed a ΔGbind of −56.33 kcal/mol, significantly higher (less favorable) than the selected compounds (Supplementary Table S6). 

## 3. Discussion

Our study examined the binding affinity, stability, and pharmacokinetic properties of three candidate compounds against the VP39 protein, a critical component in the monkeypox virus (MPXV) replication process. The analysis involved virtual screening, molecular docking, ADME profiling, molecular dynamics (MD) simulations, and free energy calculations using a control compound as a baseline [30]. By integrating these methodologies, we aimed to identify compounds with strong binding potential and favorable pharmacokinetics that could serve as antiviral agents. The approach and findings of our study align with and expand upon previous research targeting viral proteins, such as those within the poxvirus family, which includes MPXV, smallpox, and other zoonotic diseases. This discussion highlights the relevance of each method, aligns our findings with supporting studies, and addresses potential limitations while suggesting future directions. The initial screening and docking phases were instrumental in identifying high-affinity compounds [31]. We used virtual screening of the Diverse Lib library to evaluate potential inhibitors of VP39, narrowing them down to three candidates with promising docking scores. Compound **2** exhibited the highest binding affinity, slightly surpassing compounds **1** and **3**, with all three outperforming the control. The effectiveness of virtual screening for viral targets is well-supported by previous research. For instance, Vishwesh Venkatraman et al. (2024) demonstrated the efficacy of structure-based virtual screening in identifying high-affinity inhibitors for viral proteins using similar computational techniques, highlighting its value for identifying initial leads in the drug discovery procedurally, Gregory Sliwoski et al. (2014) applied docking to target SARS-CoV-2 viral proteins, successfully identifying strong binders, which further validates the use of docking scores as a predictor of binding strength in antiviral studies [30,32]. This supports our results, where the docking scores for the selected compounds indicated promising potential for VP39 inhibition, supporting the approach’s reliability for preliminary antiviral compound selection. Analyzing intermolecular interactions is critical for understanding the nature of protein–ligand binding. Our study examined hydrogen bonds, van der Waals forces, and π-π stacking interactions to assess each compound’s binding mechanism [33]. Compound **1**, which showed balanced hydrogen bonding and hydrophobic interactions, demonstrated the highest stability in our docking simulations, while compound **2** relied more on water-mediated interactions and π-π stacking, potentially contributing to enhanced binding flexibility. Compound **3** exhibited electrostatic interactions that resulted in moderate stability. These observations align with the work by Favour Olaoye et al. (2024), which found that balanced hydrogen bonding and van der Waals forces play significant roles in stabilizing protein–ligand interactions in antiviral compounds [34]. Similarly. ADME profiling of the selected compounds revealed favorable pharmacokinetic properties, which are essential for antiviral efficacy [21]. The results indicated that all compounds had acceptable absorption, distribution, metabolism, and excretion profiles, with optimal bioavailability and metabolic stability. Our findings indicate that they could exhibit satisfactory pharmacokinetic profiles in vivo, making them promising candidates for further study. Future in vitro testing could help validate these predictions and further optimize the compounds’ ADME characteristics. Molecular dynamics (MD) simulations offered insights into the structural stability and flexibility of each protein–ligand complex [35,36]. RMSD analysis indicated that compounds **1** and **2** maintained stable conformations throughout the simulation, with protein RMSD values around **2** Å, which reflects minimal deviation and suggests a strong binding interaction. In contrast, the control compound exhibited greater RMSD fluctuations, indicating a more flexible interaction with the protein binding site. RMSF analysis showed that compound **2** restricted protein flexibility more effectively than the control and compound **3**, aligning with the studies of Sharma et al. (2021), who found that compounds with stable RMSD and RMSF profiles tend to exhibit higher binding stability and antiviral efficacy. This reinforces our results, suggesting that RMSF profiles are strong indicators of binding stability and are valuable in predicting antiviral efficacy. SASA analysis, which measures the solvent exposure of each compound within the binding pocket, revealed that compound **1** became more buried over time, indicating strong binding, while compound **2** initially showed higher solvent exposure before stabilizing in the binding pocket, suggesting a flexible but stable interaction. Compound **3** displayed moderate SASA values, while the control compound had consistently low SASA, indicating a deeply buried but potentially less compact binding mode. In terms of compactness [37], Rg analysis showed that the compound exhibited low and stable Rg values, indicating structural integrity and compactness, whereas the control compound had higher Rg values, suggesting a more flexible complex. These results align with research showing that low Rg values are indicative of stable protein–ligand complexes, further supporting our conclusion that compounds **1** and **2** exhibit strong binding stability. Free binding energy calcu MM/GBSA provided quantitative insights into the binding affinities of each compound [38], with lower ΔGbind values indicating stronger binding. Compound **1** exhibited the most favorable binding energy (−87.84 kcal/mol), surpassing the control compound, which had a slightly weaker ΔGbind. This trend is consistent with findings from studies by Homeyer and Gohlke (2012), who found that MM/GBSA calculations are reliable indicators of binding affinity, with lower ΔGbind values corresponding to stronger and more stable protein–ligand interactions in antiviral research. Our results support the notion that MM/GBSA-based calculations are valuable fng docking and MD results, strengthening the case for compounds **1** and **2** as promising antiviral candidates. While our study provides valuable insights into potential VP39 inhibitors, limitations include the reliance on computational predictions without experimental validation. In silico methods are powerful tools for early-stage research but lack the biological complexity found in vivo. For example, real-world factors like cellular uptake, toxicity, and immune response can influence the efficacy and safety of compounds, which computational models cannot fully predict. Therefore, in vitro and in vivo studies are essential to confirm our findings and identify any unforeseen challenges in clinical development. Future studies could focus on validating these predictions through cell-based assays and in vivo studies to confirm efficacy and safety. Exploring the structural analogs of compounds **1** and **2** with optimized ADME properties could further enhance their antiviral potential. Additionally, investigating similar viral proteins across the poxvirus family could broaden the therapeutic range of these compounds, offering potential treatment options against other related viruses. Given the rapid evolution of viral strains, it would be beneficial to assess the compounds’ binding effectiveness against potential mutations in the VP39 protein to ensure continued efficacy. Finally, integrating machine learning approaches for predictive modeling could further refine compound selection and streamline future antiviral drug discovery efforts.

## 4. Materials and Methods

### 4.1. Data Collection

This study used computational methodology to predict potential monkeypox virus methyltransferase VP39 inhibitors. The VP39 protein structure (PDB ID: 8CEQ) was searched and downloaded for a crystal structure of high resolution to perform adequate docking studies [15,39]. The Diverse Lib database was used to gather ligand data because it presents a wide array of bioactive compounds that are well documented for their biological properties.

### 4.2. Virtual Screening and Molecular Docking

For virtual screening, the MtiOpenScreen web server was utilized [17]. Some of these compounds obtained from screening the Diverse Lib ligand library were subjected to Lipinski filters to check whether the compounds possessed drug-like features. Compounds that passed this filter underwent redocking to the VP39 protein, and three that yielded the highest scores were taken for further analysis. The redocking of the VP39 protein was done through the Chimera software by the Dock Prep tool [40]. While performing this, non-standard groups like ligands and water molecules were stripped off to minimize bias. Protein was protonated, all hydrogen atoms were placed in the protein structure, and the hydrogen bond was minimized.

The UF9 molecule present in the crystal structure of the target protein was used as the control. The correct charges were given to the amino acids according to the physiological pH for the protein, and then energy minimization was performed to discard any steric hindrance, which was performed to obtain a single protein structure ready for the process of redocking, which was performed along with the control molecule through AutoDock Vina Chimera plugin [41].

### 4.3. Dynamical Analysis

MD simulation was performed to analyze the system’s stability, dynamics, and interactions between VP39 protein and compounds [42,43]. Three of the protein–ligand complexes chosen from the Diverse Lib library by virtual screening and a control system composed of the VP39 protein and native ligand were included in the MD simulations. The simulations for each complex were carried out by Desmond-Maestro 2020-4 [40], an academic free software from Schrödinger, LLC [41], to obtain information about the binding stability and conformational behavior of the protein–ligand complexes at physiological temperature. All three systems were set up for MD simulations through the system setup Wizard in the Maestro software. This process involved the following steps to achieve the desired goal of making the systems simulation ready. Afterward, hydrogen atoms were included in the protein and ligands to make the correct protonation state at physiological pH 7.4. Lastly, if gaps on the side chains or loops were not observed in the crystal structure, they were built in by a Swiss model web server [44]. The hydrogen bond network was rebuilt to stabilize the system further. The obtained protein–ligand complex systems were solvated in a cubic box of TIP3P water molecules, consisting of a 10 Å box size [45]. The development of sodium and chloride ions allowed for the neutralization of the system and brought the value close to approximate physiological ranges in concentrations at 0. 15 M NaCl. The systems were then energy minimized using the OPLS_2005 force field to reduce unfavorable interactions or steric hindrance [46]. Following this step, the systems were subjected to several rounds of energy minimization to obtain stable conditions to begin production runs. The production MD simulations were performed for 1000 ns for each system under the NPT ensemble (Number of particles, pressure, and temperature). The simulation was performed with a Nose–Hoover thermostat at 300 K while the Martyna–Tobias–Klein barostat was applied to maintain the pressure at 1 atm [47]. The Particle Mesh Ewald (PME) was applied to calculate the long-range electrostatic interactions with a time step of 2 fs [48]. The dynamic studies were carried out using the trajectory data generated from the MD simulations to infer the stability of the VP39–ligand complexes. RMSD was used to measure the average structural change in protein throughout the simulation, and the RMSF measured the variability of residues. SASA and Rg were also calculated to determine alterations in the protein’s compactness and surface exposure. This analysis allowed us to understand the interactions that could help stabilize the VP39–ligand complexes, providing insights into the inhibitory processes of the chosen agents according to the data simulations.

### 4.4. Binding Free Energy

Molecular mechanics generalized born surface area is an effective computational approach used to calculate the binding free energy of any ligand and protein complex [38]. In the present work, the MMGBSA calculations were performed using Schrodinger Prime module with the binding enthalpy and entropy of various protein–ligand complexes [49]. This approach employs molecular mechanics to describe a given ligand–protein interaction and quantifies the free energy change upon the binding process. The output linked to this method is the relative binding free energy, referred to as ΔGbind for the ligands of interest. Free binding energies were predicted by using the MM/GBSA method on representative structures selected from the last 50 ns of the molecular dynamic simulation. A single-trajectory protocol was used to enhance the computational speed employing the GB-OBC2 model with the default atomic radii and surface tension coefficient of 0.0072 kcal/mol/Å^2^ for non-polar solvation. The other parameters include setting dielectric constants to 1 for the protein interior and 80 for the aqueous exterior, while the salt value was 0.15 M and entropic values were disregarded to compare relative binding energies of the ligands [50,51]. For the calculation of MMGBSA following equation was used:

ΔG_binding_ = ΔE_MM_ + ΔG_solvation_ − TΔS

where

ΔEMM is the molecular mechanics energy, which includes electrostatic and van der Waals interactions.
ΔGsolvation is the solvation free energy, typically comprising polar and non-polar contributions.
TΔS is the entropic contribution, often approximated or derived from normal mode analysis in certain cases.

Together, they describe different aspects of the ligand–protein interaction; in MM/GBSA binding free energy calculations, they clarify the amount of binding stability involved from various components. ΔGbind is the sum energy change upon ligand binding, so more negative energy means the ligand is stronger binding. Of these components, ΔGbind Coulomb is the component reflecting electrostatic interactions of the ligand and protein; charge-based attractions aid in stabilization of the binding. The covalent bonding aspects of ΔGbind covalent are normally expressed as small conformational changes without true bond formation, yet can still be quite impressive computationally. ΔGbind Hbond quantifies the hydrogens bonding stabilizing the complex by direct hydrogen bonding. Lipophilic (hydrophobic) interactions are captured by the ΔGbind Lipo term, which confers stability by inhibiting the water solubility of the ligand and protein hydrophobic regions. Moreover, ΔGbind packing reflects the steric packing efficiency focusing on van der Waals interactions and shape complementarity. Solvation effects are accounted for, including the energetic effect of desolvating the ligand and protein as they bind, based on the ΔGbind Solv GB term, computed with the generalized born (GB) model. vdW represents van der Waals forces and contributes to stabilization by temporary dipoles and dispersion effects. Ligand strain energy is finally a description of any strain in the ligand as it assumes a binding competent conformation and can suggest low binding stability from a large effect on structure via bind. These components together yield a detailed view of the forces responsible for ligand binding affinity.

## 5. Conclusions

This work provides a valuable analysis of the biophysical and thermodynamic characteristics of the conformational changes and binding of three selected compounds and one control compound to the VP39 protein. The methodology involved virtual screening, molecular redocking, intermolecular interaction analyses, molecular dynamics simulations and energy estimations to assess these compounds’ efficacy as inhibitors. Virtual screening and redocking analysis indicated that both compounds **1** and **2** interacted favorably with the target protein o, and compound **2** possessed the maximum binding energy value. The intermolecular interaction analyses of both compounds have indicated that while compound **1** forms strong and stable hydrogen bonds and hydrophobic interactions with the protein residues, compound **2** has more water-mediated interactions and more π-π stacking interactions, which depict the different modes of binding of the compounds. In the case of compound **3**, a different mode of binding was revealed where this compound contributed to the formation of some structural features with the help of ionic interactions and hydrophobic contacts and, therefore, could reveal different electrostatic interactions in the binding pocket. From the MD simulation, it was evident that RMSD for both the ligands, compound **1** and compound **2**, had slight fluctuation, indicating that the two ligands had good binding interaction with the target protein and were energetically stable. However, compound **3** possesses moderate flexibility. These results were also confirmed by RMSF, SASA, and radius of gyration analysis, where compounds **1** and **2** bind to protein conformation with the most stability and compactness. Moreover, substantial details concerning the binding free energies were gathered through the MM/GBSA, which established that compounds **1** and **2** exhibited higher binding affinities than compound **3**. In this case, two compounds, **1** and **2**, could be considered potential inhibitors of the VP39 protein because their binding is strong and stable, similar to other works about inhibiting viral proteins. From these findings, this research posits that these compounds may be used to develop effectual antiviral medicines for monkeypox.

## Figures and Tables

**Table 1:** Intermolecular analysis of selected compounds in the docket pocket of target protein.

Sr. no.	Complex	H-Bond	Van der Waals	π-π Stacking/ π-π Cation	Structure
1	8CEQ_17444176 (PubChem SID 17444176)	--	Lys^142^, Arg^97^, Gly^68^, Gly^96^, Asp^95^, Ser^141^, Phe^115^, Ile^94^, Ile^67^, Val^116^ Leu^159^	--	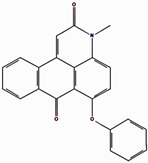
2	8CEQ_17450998 (PubChem SID 17450998)	Gly^68^,	Val^116^, Leu^159^, Asn^156^, Ser^141^, Asp^138^, Ser^69^, Gln^39^, Ile^75^, Pro^71^, Gly^72^, Ala^70^, Lys^142^, Gly^145^	Phe^115^	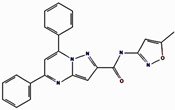
3	8CEQ_24392109 (PubChem SID 24392109)	Arg^97^, Asp^95^, Arg^140^,	Leu^42^, Asn^146^, Lys^175^, Lys^142^, Asp^138^, Gly^96^, Ile^94^, Ile^67^, Val^116^, Leu^159^, Arg^143^, Ala^70^, Ser^69^, Gly^145^	Phe^115^	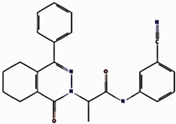
4	8CEQ_Control	Val^116^, Asp^95^, Arg^97^, Asp^138^, Gly^68^, His^74^, Gly^72^, Gln^39^	Asn^156^, Ser^141^, Ser^155^, Ser^69^, Tyr^66^, Arg^140^, Arg^114^, Ile^94^, Ile^67^, Leu^42^, Ile^75^	--	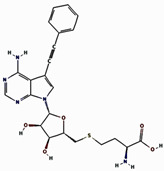

**Table 2:** MMGBSA analysis of selected compounds in the docked pocket of protein.

	17444176	17450998	24392109	Control
ΔGBind	−87.84 ± 6.45	−69.06 ± 4.84	−59.55 ± 3.99	−76.46 ± 4.89
ΔGBind Coulomb	−36.93 ± 6.42	−11.27 ± 2.76	−14.64 ± 4.47	−10.49 ± 3.53
ΔGBind Covalent	1.79 ± 1.41	4.85 ± 1.99	−0.18 ± 1.04	−0.16 ± 0.68
ΔGBind Hbond	−4.19 ± 0.81	−1.12 ± 0.37	−1.21 ± 0.39	−025 ± 0.24
ΔGBind Lipo	−19.88 ± 1.16	−15.31 ± 1.50	−19.70 ± 1.45	−27.97 ± 1.65
ΔGBind Packing	−1.22 ± 0.43	−7.06 ± 0.72	−3.60 ± 0.60	−4.72 ± 0.89
ΔGBind Solv GB	37.36 ± 4.79	20.92 ± 1.84	27.49 ± 3.17	19.70 ± 1.70
ΔGBind vdW	−64.77 ± 3.40	−60.04 ± 3.27	−47.70 ± 3.08	−52.55 ± 2.34
Ligand Strain Energy	5.45 ± 2.34	4.41 ± 1.57	5.54 ± 1.02	1.61 ± 0.51

## Data Availability

The original contributions presented in the study are included in the article/Supplementary Materials, further inquiries can be directed to the corresponding authors.

## Supplementary Materials

The following supporting information can be downloaded at: https://www.mdpi.com/article/10.3390/ph17121617/s1, Figure S1: 3D and 2D structure analysis of viral and host protein (a,b) 4QWO_17444176, (c,d) 4QWO_17450998; Figure S2: 3D and 2D structure analysis of viral and host protein (a,b) 8HTS_17444176, (c,d) 8HTS_17450998; Figure S3: 2D and 3D molecular interaction plot of negative control compound; Figure S4: RMSD AND RMSF analysis of negative control compound; Figure S5: Ligand RMSF analysis of negative control compound i.e., (a) 22406971; Figure S6: Protein-ligand profiling of Negative control; Figure S7: 2D Protein-ligand interaction analysis of Negative control; Figure S8: SASA analysis of Negative control; Figure S9: RG analysis of Negative control; Table S1: List of selected compounds used in the virtual screening; Table S2: Intermolecular analysis of viral and host protein 1- 4QWO_17444176, and 2-4QWO_17450998; Table S3: Intermolecular analysis of viral and host protein 1. 8HTS_17444176, 2. 8HTS_17450998; Table S4: MMGBSA analysis of replica 2 for selected compounds in the docked pocket of protein; Table S5: MMGBSA analysis of replica 3 for selected compounds in the docked pocket of protein; Table S6: MMGBSA analysis of Negative control.
