# Supplementary material for: Evaluating the Binding Potential and Stability of Drug-like Compounds with the Monkeypox Virus VP39 Protein Using Molecular Dynamics Simulations and Free Energy Analysis
Source: Pharmaceuticals (Basel). 2024 Nov 30;17(12):1617. doi: 10.3390/ph17121617 (PMC11728677; doi:10.3390/ph17121617)
Supplement: Supplementary file 1 [file pharmaceuticals-17-01617-s001.zip › pharmaceuticals-3228867-supplementary.pdf]

S.no	Compound	Energy
1	17444176_Intermediate	-10.8
2	17450998_Accepted	-10.6
3	24392109_Intermediate	-10.2
4	92764563_Intermediate	-10.2
5	24837488_Intermediate	-10.2
6	14741401_Intermediate	-10.1
7	24370709_Intermediate	-10
8	57264996_Intermediate	-9.9
9	14729385_Intermediate	-9.9
10	124948503_Accepted	-9.9
11	24337900_Intermediate	-9.8
12	24294582_Accepted	-9.8
13	49822165_Intermediate	-9.8
14	3713401_Intermediate	-9.8
15	24279655_Intermediate	-9.8
16	57262028_Accepted	-9.8
17	49666982_Intermediate	-9.8
18	4254964_Intermediate	-9.8
19	124948360_Accepted	-9.8
20	24837121_Accepted	-9.8
21	85271617_Intermediate	-9.8
22	49828895_Intermediate	-9.8
23	26613860_Accepted	-9.8
24	3712274_Intermediate	-9.7
25	56316263_Intermediate	-9.7
26	17437578_Intermediate	-9.7
27	7977938_Intermediate	-9.7
28	24799563_Intermediate	-9.7
29	26649370_Intermediate	-9.7
30	26663782_Accepted	-9.7
31	26621269_Intermediate	-9.7
32	24833484_Intermediate	-9.7
33	49676871_Intermediate	-9.7
34	7971721_Intermediate	-9.7
35	24832596_Intermediate	-9.7
36	26647121_Intermediate	-9.7
37	85272446_Intermediate	-9.6
38	3711514_Intermediate	-9.6
39	24279610_Intermediate	-9.6
40	26620814_Intermediate	-9.6
41	124755885_Intermediate	-9.6
42	26617995_Intermediate	-9.6

43	848721_Intermediate	-9.6
44	16953805_Intermediate	-9.6
45	47196360_Intermediate	-9.6
46	26647294_Intermediate	-9.6
47	4261397_Accepted	-9.5
48	57259006_Intermediate	-9.5
49	24836848_Intermediate	-9.5
50	24370346_Intermediate	-9.5
51	26618374_Accepted	-9.5
52	57256686_Intermediate	-9.5
53	26666250_Intermediate	-9.5
54	22409754_Intermediate	-9.5
55	26736133_Intermediate	-9.5
56	26646649_Accepted	-9.5
57	4256142_Intermediate	-9.5
58	26649657_Accepted	-9.5
59	3717068_Intermediate	-9.5
60	26616677_Intermediate	-9.5
61	14729120_Accepted	-9.5
62	136889484_Intermediate	-9.5
63	24830570_Intermediate	-9.5
64	24412722_Intermediate	-9.5
65	14737327_Intermediate	-9.5
66	57261168_Intermediate	-9.5
67	26646684_Accepted	-9.5
68	24295690_Intermediate	-9.5
69	49719029_Accepted	-9.4
70	26617977_Accepted	-9.4
71	103076299_Intermediate	-9.4
72	24799150_Intermediate	-9.4
73	843985_Intermediate	-9.4
74	29216802_Accepted	-9.4
75	121285581_Intermediate	-9.4
76	26539575_Intermediate	-9.4
77	49719655_Accepted	-9.4
78	24304677_Intermediate	-9.4
79	864178_Accepted	-9.4
80	124947978_Intermediate	-9.4
81	24406151_Intermediate	-9.4
82	17514560_Intermediate	-9.4
83	24416605_Intermediate	-9.4
84	26647727_Intermediate	-9.4
85	124756832_Intermediate	-9.4

86	16952870_Intermediate	-9.4
87	29216465_Intermediate	-9.4
88	103074952_Intermediate	-9.4
89	57256402_Intermediate	-9.4
90	24374666_Intermediate	-9.4
91	26646639_Accepted	-9.4
92	14742393_Intermediate	-9.4
93	56315692_Accepted	-9.4
94	14723690_Accepted	-9.4
95	26669962_Intermediate	-9.3
96	24802573_Intermediate	-9.3
97	865526_Accepted	-9.3
98	160654180_Intermediate	-9.3
99	26531978_Intermediate	-9.3
100	26661146_Accepted	-9.3
101	24377599_Intermediate	-9.3
102	7969046_Accepted	-9.3
103	24272815_Intermediate	-9.3
104	24784444_Intermediate	-9.3
105	49719142_Intermediate	-9.3
106	24339378_Intermediate	-9.3
107	26540357_Intermediate	-9.3
108	24373538_Intermediate	-9.3
109	7968879_Intermediate	-9.3
110	26669565_Intermediate	-9.3
111	24781712_Intermediate	-9.3
112	14725247_Intermediate	-9.3
113	4259085_Intermediate	-9.3
114	24820136_Intermediate	-9.3
115	124948768_Intermediate	-9.3
116	4241239_Intermediate	-9.3
117	85147430_Intermediate	-9.3
118	7965915_Accepted	-9.3
119	24332887_Intermediate	-9.3
120	4242752_Intermediate	-9.3
121	26649876_Intermediate	-9.3
122	14742073_Accepted	-9.3
123	87342269_Intermediate	-9.3
124	17458391_Accepted	-9.3
125	124948536_Accepted	-9.3
126	26669935_Intermediate	-9.3
127	24799915_Intermediate	-9.3
128	24822837_Intermediate	-9.3

129	49823190_Intermediate	-9.3
130	49736898_Intermediate	-9.3
131	85198802_Intermediate	-9.3
132	144099341_Intermediate	-9.3
133	24402962_Accepted	-9.3
134	17431512_Intermediate	-9.3
135	14744798_Intermediate	-9.3
136	17416032_Intermediate	-9.3
137	121283528_Accepted	-9.3
138	17453307_Intermediate	-9.3
139	14726733_Intermediate	-9.3
140	26727690_Intermediate	-9.3
141	24781276_Intermediate	-9.3
142	26662838_Accepted	-9.3
143	103163258_Accepted	-9.3
144	49674295_Intermediate	-9.2
145	26617170_Accepted	-9.2
146	57263503_Intermediate	-9.2
147	26641090_Intermediate	-9.2
148	144089635_Intermediate	-9.2
149	17468676_Accepted	-9.2
150	103074593_Intermediate	-9.2
151	22410920_Accepted	-9.2
152	85148397_Intermediate	-9.2
153	49734916_Intermediate	-9.2
154	24816218_Intermediate	-9.2
155	17517385_Intermediate	-9.2
156	49718523_Accepted	-9.2
157	99495342_Intermediate	-9.2
158	17469024_Intermediate	-9.2
159	4265044_Intermediate	-9.2
160	51089199_Intermediate	-9.2
161	14737244_Intermediate	-9.2
162	4256034_Intermediate	-9.2
163	99359271_Intermediate	-9.2
164	24397681_Intermediate	-9.2
165	26646777_Accepted	-9.2
166	26651201_Accepted	-9.2
167	865112_Intermediate	-9.2
168	24322547_Intermediate	-9.2
169	24394657_Intermediate	-9.2
170	864908_Accepted	-9.2
171	24303683_Intermediate	-9.2

172	49819585_Intermediate	-9.2
173	24374684_Intermediate	-9.2
174	17514202_Intermediate	-9.2
175	51087031_Intermediate	-9.2
176	46500320_Accepted	-9.2
177	24281222_Intermediate	-9.2
178	24416760_Intermediate	-9.2
179	14729669_Intermediate	-9.2
180	26659066_Intermediate	-9.2
181	22409111_Intermediate	-9.2
182	7966574_Intermediate	-9.2
183	22416631_Accepted	-9.2
184	49720215_Intermediate	-9.2
185	4258627_Intermediate	-9.2
186	124948465_Accepted	-9.2
187	124349937_Intermediate	-9.2
188	22415330_Intermediate	-9.2
189	124948855_Intermediate	-9.2
190	24375853_Intermediate	-9.1
191	24317634_Intermediate	-9.1
192	17505078_Accepted	-9.1
193	49826377_Intermediate	-9.1
194	17452951_Intermediate	-9.1
195	14743346_Accepted	-9.1
196	26617074_Intermediate	-9.1
197	24395365_Intermediate	-9.1
198	26650735_Accepted	-9.1
199	7965454_Intermediate	-9.1
200	24409221_Intermediate	-9.1
201	24793945_Accepted	-9.1
202	57264731_Accepted	-9.1
203	26615242_Intermediate	-9.1
204	24781231_Intermediate	-9.1
205	26535105_Intermediate	-9.1
206	24708097_Intermediate	-9.1
207	26614318_Intermediate	-9.1
208	49678612_Intermediate	-9.1
209	17440604_Intermediate	-9.1
210	49645556_Intermediate	-9.1
211	24819795_Accepted	-9.1
212	4247822_Intermediate	-9.1
213	26646871_Accepted	-9.1
214	17505423_Intermediate	-9.1

215	24339902_Intermediate	-9.1
216	3713483_Accepted	-9.1
217	846125_Intermediate	-9.1
218	4261300_Accepted	-9.1
219	864349_Intermediate	-9.1
220	24310300_Intermediate	-9.1
221	24827445_Intermediate	-9.1
222	29217702_Intermediate	-9.1
223	124756801_Accepted	-9.1
224	49672012_Intermediate	-9.1
225	24822289_Accepted	-9.1
226	51086883_Intermediate	-9.1
227	49828235_Intermediate	-9.1
228	26613643_Intermediate	-9.1
229	24799679_Intermediate	-9.1
230	4260164_Intermediate	-9.1
231	24781062_Intermediate	-9.1
232	24374411_Intermediate	-9.1
233	46500314_Accepted	-9.1
234	134419085_Accepted	-9.1
235	24839596_Intermediate	-9.1
236	24797813_Intermediate	-9.1
237	26530399_Intermediate	-9.1
238	99376615_Intermediate	-9.1
239	17508602_Intermediate	-9.1
240	56315070_Accepted	-9.1
241	49719206_Intermediate	-9.1
242	56324660_Intermediate	-9.1
243	49736246_Accepted	-9.1
244	49722986_Intermediate	-9.1
245	26618314_Intermediate	-9.1
246	26651264_Accepted	-9.1
247	17473784_Intermediate	-9.1
248	24392075_Intermediate	-9.1
249	57267117_Intermediate	-9.1
250	24315340_Intermediate	-9.1
251	17454693_Intermediate	-9.1
252	24331974_Intermediate	-9.1
253	24408934_Intermediate	-9.1
254	24827149_Intermediate	-9.1
255	4262253_Intermediate	-9
256	85736198_Intermediate	-9
257	49735036_Accepted	-9

258	26527823_Intermediate	-9
259	24400102_Intermediate	-9
260	26644854_Intermediate	-9
261	99495482_Intermediate	-9
262	49680689_Intermediate	-9
263	47197223_Accepted	-9
264	89850943_Intermediate	-9
265	49718583_Intermediate	-9
266	57256084_Intermediate	-9
267	56322632_Intermediate	-9
268	26613560_Intermediate	-9
269	24364238_Intermediate	-9
270	26531010_Intermediate	-9
271	26622118_Intermediate	-9
272	24820099_Intermediate	-9
273	24394961_Intermediate	-9
274	49733934_Accepted	-9
275	24800716_Accepted	-9
276	17413860_Intermediate	-9
277	26646945_Intermediate	-9
278	4244420_Intermediate	-9
279	22402032_Intermediate	-9
280	24836161_Intermediate	-9
281	49720997_Intermediate	-9
282	49721284_Intermediate	-9
283	7968165_Accepted	-9
284	103075298_Accepted	-9
285	26527857_Intermediate	-9
286	17505556_Intermediate	-9
287	49823337_Intermediate	-9
288	24370849_Intermediate	-9
289	26533870_Intermediate	-9
290	17402129_Accepted	-9
291	26645468_Intermediate	-9
292	24304623_Intermediate	-9
293	49820100_Intermediate	-9
294	4263689_Intermediate	-9
295	24798481_Intermediate	-9
296	99454486_Intermediate	-9
297	56321394_Accepted	-9
298	56422732_Intermediate	-9
299	26746694_Intermediate	-9
300	14730768_Accepted	-9

301	24790440_Accepted	-9
302	24799472_Accepted	-9
303	49818508_Accepted	-9
304	24326367_Accepted	-9
305	85199804_Intermediate	-9
306	47199487_Accepted	-9
307	26701743_Accepted	-9
308	49823253_Intermediate	-9
309	24332798_Accepted	-9
310	99359865_Intermediate	-9
311	26644816_Intermediate	-9
312	22404366_Intermediate	-9
313	26648948_Intermediate	-9
314	125001301_Accepted	-9
315	17452005_Intermediate	-9
316	49730790_Intermediate	-9
317	26539012_Accepted	-9
318	3717536_Intermediate	-9
319	49729670_Accepted	-9
320	49715059_Intermediate	-9
321	124949085_Intermediate	-9
322	49716634_Intermediate	-9
323	49732550_Accepted	-9
324	22409200_Intermediate	-9
325	24802454_Intermediate	-9
326	16953035_Intermediate	-9
327	49822174_Intermediate	-9
328	26614864_Intermediate	-9
329	26624876_Intermediate	-9
330	7998056_Intermediate	-9
331	24794889_Accepted	-9
332	124949254_Intermediate	-9
333	24396417_Intermediate	-9
334	104233114_Intermediate	-9
335	49720953_Intermediate	-9
336	864379_Accepted	-9
337	26648094_Accepted	-9
338	49724091_Intermediate	-9
339	24794555_Intermediate	-9
340	26626190_Intermediate	-8.9
341	124948103_Accepted	-8.9
342	24822505_Intermediate	-8.9
343	24305992_Intermediate	-8.9

344	124948755_Intermediate	-8.9
345	92764238_Intermediate	-8.9
346	24345972_Intermediate	-8.9
347	26618057_Accepted	-8.9
348	22401615_Intermediate	-8.9
349	24310203_Accepted	-8.9
350	17468647_Intermediate	-8.9
351	7971098_Intermediate	-8.9
352	49829297_Accepted	-8.9
353	26535024_Intermediate	-8.9
354	26647798_Intermediate	-8.9
355	121285583_Intermediate	-8.9
356	24300535_Intermediate	-8.9
357	24825630_Intermediate	-8.9
358	57288061_Intermediate	-8.9
359	24373964_Accepted	-8.9
360	49816195_Intermediate	-8.9
361	103050726_Accepted	-8.9
362	24365815_Intermediate	-8.9
363	57258695_Accepted	-8.9
364	24840485_Intermediate	-8.9
365	24396957_Intermediate	-8.9
366	49720262_Intermediate	-8.9
367	17410015_Intermediate	-8.9
368	24792865_Intermediate	-8.9
369	49829182_Intermediate	-8.9
370	137275924_Intermediate	-8.9
371	14723822_Intermediate	-8.9
372	24406717_Intermediate	-8.9
373	4243135_Intermediate	-8.9
374	49721980_Intermediate	-8.9
375	26636558_Intermediate	-8.9
376	49723533_Accepted	-8.9
377	17473379_Intermediate	-8.9
378	51086301_Intermediate	-8.9
379	7970912_Intermediate	-8.9
380	17450594_Accepted	-8.9
381	24781368_Accepted	-8.9
382	99359897_Intermediate	-8.9
383	3716856_Accepted	-8.9
384	7976272_Intermediate	-8.9
385	24789184_Intermediate	-8.9
386	861829_Accepted	-8.9

387	7969145_Intermediate	-8.9
388	49723320_Intermediate	-8.9
389	89854531_Intermediate	-8.9
390	24831842_Accepted	-8.9
391	57266468_Intermediate	-8.9
392	26538851_Intermediate	-8.9
393	4259916_Intermediate	-8.9
394	26649337_Accepted	-8.9
395	26649887_Intermediate	-8.9
396	24368243_Intermediate	-8.9
397	26651276_Accepted	-8.9
398	87550855_Intermediate	-8.9
399	57266632_Accepted	-8.9
400	49676385_Intermediate	-8.9
401	24391048_Intermediate	-8.9
402	49828805_Accepted	-8.9
403	17465009_Intermediate	-8.9
404	49649675_Intermediate	-8.9
405	49642071_Intermediate	-8.9
406	49828567_Intermediate	-8.9
407	24330502_Accepted	-8.9
408	17477261_Intermediate	-8.9
409	24270506_Intermediate	-8.9
410	7967725_Intermediate	-8.9
411	49732641_Accepted	-8.9
412	49671251_Accepted	-8.9
413	3712179_Intermediate	-8.9
414	3713016_Accepted	-8.9
415	24326753_Intermediate	-8.9
416	57258466_Intermediate	-8.9
417	24799483_Intermediate	-8.9
418	26661533_Accepted	-8.9
419	49736712_Intermediate	-8.9
420	26618187_Intermediate	-8.9
421	24324319_Accepted	-8.9
422	26633101_Intermediate	-8.9
423	26647931_Accepted	-8.9
424	4244985_Accepted	-8.9
425	49731085_Intermediate	-8.9
426	124755881_Intermediate	-8.9
427	47201501_Intermediate	-8.9
428	57264794_Intermediate	-8.9
429	24309093_Intermediate	-8.9

430	103073652_Intermediate	-8.9
431	110167687_Intermediate	-8.9
432	57261439_Intermediate	-8.9
433	4262402_Intermediate	-8.9
434	24399599_Intermediate	-8.9
435	26648949_Intermediate	-8.9
436	49718495_Accepted	-8.9
437	85285522_Intermediate	-8.9
438	7977871_Intermediate	-8.9
439	49720503_Accepted	-8.9
440	24815672_Accepted	-8.9
441	24429114_Accepted	-8.9
442	26653280_Intermediate	-8.9
443	14746664_Intermediate	-8.9
444	92763814_Intermediate	-8.9
445	866154_Intermediate	-8.9
446	26651438_Intermediate	-8.9
447	49648862_Intermediate	-8.9
448	26647787_Intermediate	-8.9
449	26615356_Intermediate	-8.9
450	49649234_Intermediate	-8.9
451	24808018_Intermediate	-8.9
452	24395668_Intermediate	-8.9
453	49821021_Intermediate	-8.9
454	24396956_Intermediate	-8.9
455	24818305_Intermediate	-8.9
456	49645532_Accepted	-8.8
457	51085967_Intermediate	-8.8
458	56323402_Intermediate	-8.8
459	24322996_Intermediate	-8.8
460	49828307_Intermediate	-8.8
461	14735625_Intermediate	-8.8
462	24828790_Accepted	-8.8
463	99495345_Intermediate	-8.8
464	26651137_Intermediate	-8.8
465	17432374_Intermediate	-8.8
466	47201215_Intermediate	-8.8
467	24406157_Intermediate	-8.8
468	24365300_Intermediate	-8.8
469	124948115_Accepted	-8.8
470	85199023_Accepted	-8.8
471	49719212_Intermediate	-8.8
472	24410339_Intermediate	-8.8

473	49669838_Accepted	-8.8
474	26726010_Intermediate	-8.8
475	17472554_Intermediate	-8.8
476	46501006_Accepted	-8.8
477	56432202_Accepted	-8.8
478	56315354_Intermediate	-8.8
479	103074468_Intermediate	-8.8
480	26642700_Intermediate	-8.8
481	14741311_Intermediate	-8.8
482	56318192_Accepted	-8.8
483	125305729_Intermediate	-8.8
484	89853997_Intermediate	-8.8
485	14734414_Intermediate	-8.8
486	14737315_Intermediate	-8.8
487	24836894_Intermediate	-8.8
488	49648217_Accepted	-8.8
489	7965961_Accepted	-8.8
490	24407405_Intermediate	-8.8
491	26647082_Accepted	-8.8
492	26538353_Intermediate	-8.8
493	56324969_Intermediate	-8.8
494	29216503_Accepted	-8.8
495	24392153_Intermediate	-8.8
496	24825399_Accepted	-8.8
497	85148315_Intermediate	-8.8
498	7970748_Intermediate	-8.8
499	85267972_Intermediate	-8.8
500	49672150_Intermediate	-8.8
501	26729957_Intermediate	-8.8
502	17385841_Intermediate	-8.8
503	24369068_Accepted	-8.8
504	49720551_Accepted	-8.8
505	49826544_Accepted	-8.8
506	4265463_Accepted	-8.8
507	47204804_Intermediate	-8.8
508	57259056_Intermediate	-8.8
509	125306473_Accepted	-8.8
510	24798477_Intermediate	-8.8
511	24359206_Accepted	-8.8
512	24363703_Intermediate	-8.8
513	26646580_Intermediate	-8.8
514	26647560_Intermediate	-8.8
515	24391476_Accepted	-8.8

516	49721256_Accepted	-8.8
517	17438706_Intermediate	-8.8
518	843150_Intermediate	-8.8
519	99359899_Intermediate	-8.8
520	56319292_Intermediate	-8.8
521	49736537_Intermediate	-8.8
522	24328366_Accepted	-8.8
523	136889460_Intermediate	-8.8
524	24815625_Intermediate	-8.8
525	24295549_Accepted	-8.8
526	24405043_Intermediate	-8.8
527	24836219_Intermediate	-8.8
528	49725830_Intermediate	-8.8
529	24802245_Intermediate	-8.8
530	85198424_Intermediate	-8.8
531	24812777_Intermediate	-8.8
532	14733523_Intermediate	-8.8
533	22407797_Accepted	-8.8
534	26669796_Accepted	-8.8
535	89855951_Intermediate	-8.8
536	26540451_Intermediate	-8.8
537	26664833_Intermediate	-8.8
538	103050752_Accepted	-8.8
539	24329511_Intermediate	-8.8
540	24808331_Intermediate	-8.8
541	144206773_Intermediate	-8.8
542	14738841_Intermediate	-8.8
543	124349919_Intermediate	-8.8
544	26529288_Intermediate	-8.8
545	846938_Intermediate	-8.8
546	3717961_Accepted	-8.8
547	24343338_Intermediate	-8.8
548	49821756_Intermediate	-8.8
549	14731936_Intermediate	-8.8
550	26649838_Intermediate	-8.8
551	49680492_Accepted	-8.8
552	56423139_Intermediate	-8.8
553	26614559_Intermediate	-8.8
554	26620053_Intermediate	-8.8
555	14719123_Intermediate	-8.8
556	49818734_Accepted	-8.8
557	56373759_Intermediate	-8.8
558	26731542_Intermediate	-8.8

559	99495220_Intermediate	-8.8
560	26724238_Intermediate	-8.8
561	56317730_Intermediate	-8.8
562	14736138_Intermediate	-8.8
563	17466500_Intermediate	-8.8
564	24796652_Intermediate	-8.8
565	26658304_Intermediate	-8.8
566	49729009_Intermediate	-8.8
567	26538604_Intermediate	-8.8
568	57262097_Accepted	-8.8
569	26630120_Intermediate	-8.8
570	26626892_Intermediate	-8.8
571	24399112_Intermediate	-8.8
572	26728751_Intermediate	-8.8
573	56321589_Intermediate	-8.8
574	49720940_Intermediate	-8.8
575	49827802_Intermediate	-8.8
576	26661185_Intermediate	-8.8
577	26530807_Accepted	-8.8
578	49817901_Intermediate	-8.8
579	49724673_Intermediate	-8.8
580	47195894_Accepted	-8.8
581	49676185_Accepted	-8.7
582	24411791_Accepted	-8.7
583	26649564_Accepted	-8.7
584	49675231_Accepted	-8.7
585	22434098_Intermediate	-8.7
586	57265246_Intermediate	-8.7
587	144095453_Intermediate	-8.7
588	26650314_Intermediate	-8.7
589	24810362_Accepted	-8.7
590	24799977_Accepted	-8.7
591	3714943_Accepted	-8.7
592	24270746_Intermediate	-8.7
593	56324401_Accepted	-8.7
594	49819343_Intermediate	-8.7
595	22408697_Intermediate	-8.7
596	3713655_Accepted	-8.7
597	26645160_Intermediate	-8.7
598	7969278_Intermediate	-8.7
599	85149065_Accepted	-8.7
600	4265515_Accepted	-8.7
601	24788372_Intermediate	-8.7

602	49719461_Intermediate	-8.7
603	17474869_Intermediate	-8.7
604	14733838_Accepted	-8.7
605	49734732_Intermediate	-8.7
606	49721181_Accepted	-8.7
607	22407657_Intermediate	-8.7
608	4247908_Accepted	-8.7
609	24825492_Intermediate	-8.7
610	47200400_Intermediate	-8.7
611	26664391_Accepted	-8.7
612	14738426_Intermediate	-8.7
613	49673001_Accepted	-8.7
614	24289091_Intermediate	-8.7
615	24417027_Intermediate	-8.7
616	144091837_Intermediate	-8.7
617	4251092_Intermediate	-8.7
618	49723997_Intermediate	-8.7
619	57261708_Intermediate	-8.7
620	26729381_Accepted	-8.7
621	49718462_Intermediate	-8.7
622	24802124_Intermediate	-8.7
623	14741097_Accepted	-8.7
624	22410339_Intermediate	-8.7
625	57256428_Intermediate	-8.7
626	26650836_Intermediate	-8.7
627	57265796_Intermediate	-8.7
628	26648532_Accepted	-8.7
629	49674442_Intermediate	-8.7
630	57257025_Accepted	-8.7
631	7967040_Accepted	-8.7
632	49680781_Intermediate	-8.7
633	24785965_Intermediate	-8.7
634	26726186_Intermediate	-8.7
635	24331604_Accepted	-8.7
636	24829185_Intermediate	-8.7
637	17511128_Intermediate	-8.7
638	26620902_Intermediate	-8.7
639	103060821_Intermediate	-8.7
640	17385777_Intermediate	-8.7
641	24268417_Intermediate	-8.7
642	844511_Intermediate	-8.7
643	4242203_Intermediate	-8.7
644	24298618_Intermediate	-8.7

645	49817331_Intermediate	-8.7
646	4255328_Accepted	-8.7
647	49719813_Intermediate	-8.7
648	57268588_Intermediate	-8.7
649	49821439_Intermediate	-8.7
650	49732852_Intermediate	-8.7
651	24779915_Intermediate	-8.7
652	4240857_Intermediate	-8.7
653	49822181_Intermediate	-8.7
654	22405342_Intermediate	-8.7
655	49674887_Intermediate	-8.7
656	124756730_Accepted	-8.7
657	47203413_Accepted	-8.7
658	49713969_Intermediate	-8.7
659	24799211_Intermediate	-8.7
660	26649566_Intermediate	-8.7
661	26648311_Intermediate	-8.7
662	57261201_Intermediate	-8.7
663	24840208_Intermediate	-8.7
664	24395603_Intermediate	-8.7
665	4265144_Intermediate	-8.7
666	24270580_Intermediate	-8.7
667	11534909_Intermediate	-8.7
668	49641817_Intermediate	-8.7
669	24800597_Intermediate	-8.7
670	24365552_Intermediate	-8.7
671	124947971_Accepted	-8.7
672	50117369_Accepted	-8.7
673	99356493_Intermediate	-8.7
674	26730382_Intermediate	-8.7
675	14726044_Accepted	-8.7
676	24358689_Accepted	-8.7
677	103074946_Intermediate	-8.7
678	49675982_Accepted	-8.7
679	17483148_Intermediate	-8.7
680	85271110_Accepted	-8.7
681	85149117_Intermediate	-8.7
682	24798694_Intermediate	-8.7
683	24369467_Intermediate	-8.7
684	24798473_Intermediate	-8.7
685	124948290_Accepted	-8.7
686	845028_Intermediate	-8.7
687	57255727_Intermediate	-8.7

688	24410227_Intermediate	-8.7
689	26514173_Accepted	-8.7
690	26631395_Intermediate	-8.7
691	24287987_Accepted	-8.7
692	57256344_Accepted	-8.7
693	26646226_Intermediate	-8.7
694	51085470_Accepted	-8.7
695	49737498_Accepted	-8.7
696	24308998_Intermediate	-8.7
697	56318696_Intermediate	-8.7
698	49736777_Accepted	-8.7
699	26615578_Intermediate	-8.7
700	865122_Intermediate	-8.7
701	24798667_Intermediate	-8.7
702	24272823_Intermediate	-8.7
703	92764250_Accepted	-8.7
704	24409105_Intermediate	-8.7
705	24357278_Intermediate	-8.7
706	17457892_Intermediate	-8.7
707	14741682_Intermediate	-8.7
708	22402339_Accepted	-8.7
709	24786428_Intermediate	-8.7
710	57263592_Intermediate	-8.7
711	26632666_Intermediate	-8.7
712	26529701_Intermediate	-8.7
713	50100911_Intermediate	-8.7
714	24400760_Intermediate	-8.7
715	24812414_Intermediate	-8.7
716	7970936_Intermediate	-8.7
717	26538036_Intermediate	-8.7
718	7969500_Accepted	-8.7
719	49733540_Intermediate	-8.7
720	17517433_Intermediate	-8.7
721	49737701_Intermediate	-8.7
722	57257983_Intermediate	-8.7
723	844135_Accepted	-8.7
724	49644441_Accepted	-8.7
725	49820421_Intermediate	-8.7
726	99356498_Intermediate	-8.7
727	17465553_Intermediate	-8.7
728	3711570_Intermediate	-8.7
729	24835165_Intermediate	-8.7
730	56319411_Intermediate	-8.7

731	24800258_Intermediate	-8.6
732	134216222_Intermediate	-8.6
733	51086909_Accepted	-8.6
734	24818078_Intermediate	-8.6
735	24387296_Accepted	-8.6
736	57260201_Intermediate	-8.6
737	49817317_Intermediate	-8.6
738	24835083_Accepted	-8.6
739	85269900_Intermediate	-8.6
740	11114084_Accepted	-8.6
741	24373200_Accepted	-8.6
742	24357895_Intermediate	-8.6
743	24836482_Accepted	-8.6
744	26647011_Intermediate	-8.6
745	26724277_Intermediate	-8.6
746	24365255_Intermediate	-8.6
747	24811471_Intermediate	-8.6
748	24394637_Intermediate	-8.6
749	24818955_Intermediate	-8.6
750	134215449_Accepted	-8.6
751	26536312_Intermediate	-8.6
752	7975919_Accepted	-8.6
753	3713377_Intermediate	-8.6
754	26531871_Intermediate	-8.6
755	49824878_Intermediate	-8.6
756	857775_Intermediate	-8.6
757	49681582_Accepted	-8.6
758	49816520_Intermediate	-8.6
759	49680611_Intermediate	-8.6
760	56324449_Accepted	-8.6
761	85148520_Intermediate	-8.6
762	47195966_Accepted	-8.6
763	26644173_Accepted	-8.6
764	49721843_Intermediate	-8.6
765	103074830_Intermediate	-8.6
766	4249377_Intermediate	-8.6
767	49731287_Intermediate	-8.6
768	50113225_Intermediate	-8.6
769	57262944_Intermediate	-8.6
770	24275883_Intermediate	-8.6
771	11536265_Accepted	-8.6
772	24416902_Intermediate	-8.6
773	17412799_Intermediate	-8.6

774	24799160_Intermediate	-8.6
775	17474559_Accepted	-8.6
776	3714038_Intermediate	-8.6
777	26625057_Intermediate	-8.6
778	7973889_Intermediate	-8.6
779	49826116_Accepted	-8.6
780	4252108_Intermediate	-8.6
781	26650215_Intermediate	-8.6
782	26530443_Intermediate	-8.6
783	22403156_Intermediate	-8.6
784	17447879_Intermediate	-8.6
785	144090857_Intermediate	-8.6
786	50118013_Intermediate	-8.6
787	24313354_Intermediate	-8.6
788	103060406_Intermediate	-8.6
789	14724173_Intermediate	-8.6
790	24363464_Intermediate	-8.6
791	57268779_Intermediate	-8.6
792	26651075_Intermediate	-8.6
793	50111329_Intermediate	-8.6
794	24346425_Intermediate	-8.6
795	26651394_Intermediate	-8.6
796	26659826_Intermediate	-8.6
797	3714776_Intermediate	-8.6
798	85198947_Intermediate	-8.6
799	4257592_Accepted	-8.6
800	24357819_Intermediate	-8.6
801	24358186_Intermediate	-8.6
802	26616554_Intermediate	-8.6
803	17468476_Intermediate	-8.6
804	24785135_Accepted	-8.6
805	26647416_Intermediate	-8.6
806	26613755_Accepted	-8.6
807	14744050_Intermediate	-8.6
808	57260247_Intermediate	-8.6
809	24324489_Intermediate	-8.6
810	26638168_Intermediate	-8.6
811	22433972_Intermediate	-8.6
812	174006469_Intermediate	-8.6
813	26645560_Intermediate	-8.6
814	24403263_Intermediate	-8.6
815	24412759_Intermediate	-8.6
816	57261276_Accepted	-8.6

817	49677661_Intermediate	-8.6
818	85199070_Intermediate	-8.6
819	26537072_Intermediate	-8.6
820	26621375_Intermediate	-8.6
821	863511_Intermediate	-8.6
822	17475437_Intermediate	-8.6
823	17515196_Intermediate	-8.6
824	24417075_Intermediate	-8.6
825	49822194_Intermediate	-8.6
826	49731511_Intermediate	-8.6
827	26661255_Intermediate	-8.6
828	26647215_Intermediate	-8.6
829	85271518_Intermediate	-8.6
830	24394312_Intermediate	-8.6
831	26661832_Intermediate	-8.6
832	24830047_Accepted	-8.6
833	49665676_Intermediate	-8.6
834	26629621_Intermediate	-8.6
835	860877_Intermediate	-8.6
836	24785818_Intermediate	-8.6
837	49678183_Intermediate	-8.6
838	49672532_Intermediate	-8.6
839	26651165_Accepted	-8.6
840	26650470_Intermediate	-8.6
841	17504238_Intermediate	-8.6
842	57263773_Intermediate	-8.6
843	17447965_Intermediate	-8.6
844	24299785_Accepted	-8.6
845	24840714_Intermediate	-8.6
846	26615191_Intermediate	-8.6
847	14732688_Intermediate	-8.6
848	17387458_Accepted	-8.6
849	49718476_Intermediate	-8.6
850	3715142_Intermediate	-8.6
851	4257012_Accepted	-8.6
852	85736204_Accepted	-8.6
853	99357733_Accepted	-8.6
854	17475527_Intermediate	-8.6
855	24410330_Accepted	-8.6
856	90341591_Accepted	-8.6
857	858920_Intermediate	-8.6
858	7970650_Intermediate	-8.6
859	49721325_Intermediate	-8.6

860	24365251_Intermediate	-8.6
861	7967957_Intermediate	-8.6
862	24815157_Intermediate	-8.6
863	24409507_Intermediate	-8.6
864	848308_Intermediate	-8.6
865	49672485_Accepted	-8.6
866	103158879_Intermediate	-8.6
867	51085377_Intermediate	-8.6
868	26539437_Intermediate	-8.6
869	46500554_Intermediate	-8.6
870	49826880_Intermediate	-8.6
871	26534015_Accepted	-8.6
872	22400822_Intermediate	-8.6
873	85198253_Accepted	-8.6
874	57263236_Intermediate	-8.6
875	24783009_Intermediate	-8.6
876	14735606_Intermediate	-8.6
877	49648450_Accepted	-8.6
878	24818591_Intermediate	-8.6
879	85271513_Intermediate	-8.6
880	14721637_Intermediate	-8.6
881	17461010_Accepted	-8.6
882	14728006_Accepted	-8.6
883	24428931_Intermediate	-8.6
884	24841499_Intermediate	-8.6
885	4260504_Intermediate	-8.6
886	24390463_Accepted	-8.6
887	4264721_Accepted	-8.6
888	22408392_Intermediate	-8.6
889	24373929_Intermediate	-8.6
890	22412883_Intermediate	-8.6
891	49719819_Intermediate	-8.6
892	103075399_Accepted	-8.6
893	49640654_Intermediate	-8.6
894	17432937_Intermediate	-8.6
895	26648699_Intermediate	-8.6
896	51086017_Intermediate	-8.6
897	49680591_Accepted	-8.6
898	26642518_Intermediate	-8.6
899	24396124_Intermediate	-8.6
900	4244764_Intermediate	-8.6
901	104232487_Intermediate	-8.6
902	3713693_Intermediate	-8.6

903	22402415_Accepted	-8.6
904	17386237_Accepted	-8.5
905	846204_Accepted	-8.5
906	99495425_Intermediate	-8.5
907	85736215_Intermediate	-8.5
908	7967709_Intermediate	-8.5
909	24787756_Intermediate	-8.5
910	4263243_Intermediate	-8.5
911	26539753_Intermediate	-8.5
912	26646016_Intermediate	-8.5
913	17431693_Intermediate	-8.5
914	49724298_Intermediate	-8.5
915	24412370_Intermediate	-8.5
916	24318822_Intermediate	-8.5
917	17515341_Intermediate	-8.5
918	74373995_Accepted	-8.5
919	56315424_Intermediate	-8.5
920	24835078_Intermediate	-8.5
921	24793211_Accepted	-8.5
922	4248534_Intermediate	-8.5
923	103163597_Accepted	-8.5
924	49680757_Intermediate	-8.5
925	24810361_Accepted	-8.5
926	17461070_Intermediate	-8.5
927	26646638_Accepted	-8.5
928	24798384_Intermediate	-8.5
929	26614591_Intermediate	-8.5
930	24801673_Intermediate	-8.5
931	103051317_Accepted	-8.5
932	7972071_Intermediate	-8.5
933	24275642_Intermediate	-8.5
934	7975049_Intermediate	-8.5
935	49716571_Intermediate	-8.5
936	118043966_Intermediate	-8.5
937	26668961_Accepted	-8.5
938	17449738_Intermediate	-8.5
939	103913575_Accepted	-8.5
940	26666970_Intermediate	-8.5
941	92763542_Intermediate	-8.5
942	56322410_Intermediate	-8.5
943	14739094_Intermediate	-8.5
944	49818832_Intermediate	-8.5
945	26729564_Accepted	-8.5

946	26662345_Intermediate	-8.5
947	3712088_Intermediate	-8.5
948	24345125_Accepted	-8.5
949	4265126_Accepted	-8.5
950	24823275_Intermediate	-8.5
951	26613966_Intermediate	-8.5
952	26671229_Intermediate	-8.5
953	89852803_Intermediate	-8.5
954	26726478_Intermediate	-8.5
955	17443411_Accepted	-8.5
956	865283_Intermediate	-8.5
957	4264425_Intermediate	-8.5
958	56320392_Intermediate	-8.5
959	26751892_Accepted	-8.5
960	26646711_Intermediate	-8.5
961	22408818_Intermediate	-8.5
962	24822661_Intermediate	-8.5
963	853723_Intermediate	-8.5
964	24816188_Intermediate	-8.5
965	24839368_Intermediate	-8.5
966	14729545_Accepted	-8.5
967	26647732_Intermediate	-8.5
968	99454153_Intermediate	-8.5
969	17481648_Intermediate	-8.5
970	22401884_Accepted	-8.5
971	124948396_Accepted	-8.5
972	24839988_Intermediate	-8.5
973	56316296_Intermediate	-8.5
974	103075127_Intermediate	-8.5
975	24825247_Intermediate	-8.5
976	49648839_Accepted	-8.5
977	24836554_Intermediate	-8.5
978	51088534_Intermediate	-8.5
979	99456113_Intermediate	-8.5
980	51086382_Intermediate	-8.5
981	85199183_Intermediate	-8.5
982	24839351_Intermediate	-8.5
983	24831431_Intermediate	-8.5
984	57264061_Intermediate	-8.5
985	7974532_Intermediate	-8.5
986	7965624_Intermediate	-8.5
987	24786130_Intermediate	-8.5
988	17441816_Accepted	-8.5

989	57267827_Accepted	-8.5
990	24373096_Intermediate	-8.5
991	24399504_Intermediate	-8.5
992	26732407_Accepted	-8.5
993	14742793_Intermediate	-8.5
994	24332417_Intermediate	-8.5
995	14723779_Accepted	-8.5
996	24812957_Intermediate	-8.5
997	26615266_Accepted	-8.5
998	24789914_Intermediate	-8.5
999	47200688_Intermediate	-8.5
1000	24805648_Intermediate	-8.5
1001	26618260_Intermediate	-8.5
1002	104222923_Intermediate	-8.5
1003	26531473_Accepted	-8.5
1004	17412866_Intermediate	-8.5
1005	24323581_Accepted	-8.5
1006	14730209_Accepted	-8.5
1007	17408011_Intermediate	-8.5
1008	24798892_Intermediate	-8.5
1009	49647765_Accepted	-8.5
1010	24322480_Intermediate	-8.5
1011	26613523_Accepted	-8.5
1012	24819778_Intermediate	-8.5
1013	14738225_Intermediate	-8.5
1014	24780329_Intermediate	-8.5
1015	57256903_Intermediate	-8.5
1016	49667730_Intermediate	-8.5
1017	4257555_Intermediate	-8.5
1018	124349841_Intermediate	-8.5
1019	49724264_Accepted	-8.5
1020	26537498_Intermediate	-8.5
1021	26649715_Intermediate	-8.5
1022	24805094_Accepted	-8.5
1023	99431170_Intermediate	-8.5
1024	24395655_Intermediate	-8.5
1025	861924_Accepted	-8.5
1026	24787145_Intermediate	-8.5
1027	26634504_Intermediate	-8.5
1028	85149055_Intermediate	-8.5
1029	47200143_Accepted	-8.5
1030	24786156_Intermediate	-8.5
1031	26535248_Accepted	-8.5

1032	26629771_Accepted	-8.5
1033	17407580_Intermediate	-8.5
1034	49671659_Accepted	-8.5
1035	24358837_Intermediate	-8.5
1036	4245752_Intermediate	-8.5
1037	57263612_Intermediate	-8.5
1038	848239_Accepted	-8.5
1039	17517459_Intermediate	-8.5
1040	3716790_Intermediate	-8.5
1041	24810147_Accepted	-8.5
1042	16953409_Intermediate	-8.5
1043	51085706_Intermediate	-8.5
1044	24416527_Intermediate	-8.5
1045	57267730_Accepted	-8.5
1046	49672474_Intermediate	-8.5
1047	17457191_Intermediate	-8.5
1048	3717037_Intermediate	-8.5
1049	17466435_Intermediate	-8.5
1050	24791263_Intermediate	-8.5
1051	3714883_Intermediate	-8.5
1052	24291860_Intermediate	-8.5
1053	56314819_Intermediate	-8.5
1054	49677136_Intermediate	-8.5
1055	56318926_Accepted	-8.5
1056	49722138_Intermediate	-8.5
1057	26540817_Intermediate	-8.5
1058	49819423_Intermediate	-8.5
1059	24799951_Intermediate	-8.5
1060	47196829_Intermediate	-8.5
1061	49717694_Intermediate	-8.5
1062	51087715_Intermediate	-8.5
1063	24826989_Intermediate	-8.5
1064	24796567_Intermediate	-8.5
1065	99360754_Intermediate	-8.5
1066	24293964_Accepted	-8.5
1067	7964678_Intermediate	-8.5
1068	51085581_Intermediate	-8.5
1069	24825055_Intermediate	-8.5
1070	124349838_Intermediate	-8.5
1071	56324194_Intermediate	-8.5
1072	24783611_Intermediate	-8.5
1073	87344825_Accepted	-8.5
1074	24332181_Intermediate	-8.5

1075	49817722_Intermediate	-8.5
1076	14720706_Accepted	-8.5
1077	17478983_Accepted	-8.5
1078	24267120_Accepted	-8.5
1079	49829130_Intermediate	-8.5
1080	17469923_Intermediate	-8.5
1081	57260865_Intermediate	-8.5
1082	24355653_Intermediate	-8.5
1083	49714795_Intermediate	-8.5
1084	85736328_Intermediate	-8.5
1085	49822564_Intermediate	-8.5
1086	7974087_Intermediate	-8.5
1087	49735765_Intermediate	-8.5
1088	56315120_Accepted	-8.5
1089	26631972_Intermediate	-8.5
1090	49672887_Intermediate	-8.5
1091	26621703_Accepted	-8.5
1092	104169648_Intermediate	-8.5
1093	17468560_Accepted	-8.5
1094	49727154_Intermediate	-8.5
1095	3711398_Intermediate	-8.5
1096	24405548_Accepted	-8.5
1097	49680533_Intermediate	-8.5
1098	842179_Intermediate	-8.5
1099	26529452_Intermediate	-8.5
1100	17440322_Intermediate	-8.5
1101	26514328_Intermediate	-8.5
1102	24810618_Intermediate	-8.5
1103	26616065_Intermediate	-8.5
1104	24387020_Intermediate	-8.5
1105	24387750_Intermediate	-8.5
1106	26649705_Intermediate	-8.5
1107	137276044_Accepted	-8.5
1108	24409109_Intermediate	-8.5
1109	51090514_Intermediate	-8.5
1110	49722061_Accepted	-8.5
1111	144220866_Intermediate	-8.5
1112	124350480_Intermediate	-8.5
1113	26614169_Intermediate	-8.5
1114	24411853_Intermediate	-8.5
1115	47200170_Intermediate	-8.5
1116	26630764_Intermediate	-8.5
1117	26538412_Intermediate	-8.5

1118	24374063_Intermediate	-8.5
1119	49737529_Intermediate	-8.5
1120	14744389_Intermediate	-8.5
1121	49714598_Intermediate	-8.5
1122	24372071_Intermediate	-8.5
1123	17516290_Intermediate	-8.5
1124	3714511_Intermediate	-8.5
1125	3714834_Intermediate	-8.5
1126	26622256_Intermediate	-8.5
1127	24799822_Accepted	-8.4
1128	7968336_Intermediate	-8.4
1129	24300792_Intermediate	-8.4
1130	26646596_Intermediate	-8.4
1131	24352299_Intermediate	-8.4
1132	24824752_Intermediate	-8.4
1133	150864395_Accepted	-8.4
1134	49672329_Intermediate	-8.4
1135	81066380_Intermediate	-8.4
1136	4260702_Accepted	-8.4
1137	81065998_Intermediate	-8.4
1138	24820618_Intermediate	-8.4
1139	11534737_Accepted	-8.4
1140	24810496_Accepted	-8.4
1141	49825969_Intermediate	-8.4
1142	24385881_Intermediate	-8.4
1143	49732264_Intermediate	-8.4
1144	90341467_Intermediate	-8.4
1145	866129_Intermediate	-8.4
1146	26614871_Intermediate	-8.4
1147	24798608_Intermediate	-8.4
1148	3712640_Accepted	-8.4
1149	26530438_Intermediate	-8.4
1150	17386997_Intermediate	-8.4
1151	24833079_Accepted	-8.4
1152	49679425_Accepted	-8.4
1153	24405807_Intermediate	-8.4
1154	14728252_Intermediate	-8.4
1155	125306958_Accepted	-8.4
1156	17448528_Accepted	-8.4
1157	103075841_Intermediate	-8.4
1158	144098287_Intermediate	-8.4
1159	24401113_Intermediate	-8.4
1160	57264187_Intermediate	-8.4

1161	17453137_Intermediate	-8.4
1162	22411586_Intermediate	-8.4
1163	57265079_Intermediate	-8.4
1164	49734116_Intermediate	-8.4
1165	99357610_Intermediate	-8.4
1166	24387837_Intermediate	-8.4
1167	85268305_Intermediate	-8.4
1168	24781812_Intermediate	-8.4
1169	17478907_Intermediate	-8.4
1170	17387724_Accepted	-8.4
1171	24837466_Accepted	-8.4
1172	26646677_Accepted	-8.4
1173	26614675_Intermediate	-8.4
1174	49736754_Intermediate	-8.4
1175	56315429_Intermediate	-8.4
1176	22406785_Intermediate	-8.4
1177	22403301_Intermediate	-8.4
1178	4261763_Accepted	-8.4
1179	24828250_Intermediate	-8.4
1180	4247145_Intermediate	-8.4
1181	49642835_Intermediate	-8.4
1182	4264855_Intermediate	-8.4
1183	51090313_Intermediate	-8.4
1184	49675804_Accepted	-8.4
1185	848537_Accepted	-8.4
1186	47201785_Intermediate	-8.4
1187	24308805_Intermediate	-8.4
1188	49720285_Intermediate	-8.4
1189	49731692_Accepted	-8.4
1190	49829194_Intermediate	-8.4
1191	24417394_Intermediate	-8.4
1192	16953535_Intermediate	-8.4
1193	99381131_Intermediate	-8.4
1194	124949106_Intermediate	-8.4
1195	24325739_Intermediate	-8.4
1196	24797706_Intermediate	-8.4
1197	4251244_Intermediate	-8.4
1198	24832027_Intermediate	-8.4
1199	26726849_Accepted	-8.4
1200	17441234_Accepted	-8.4
1201	26617195_Accepted	-8.4
1202	24824363_Intermediate	-8.4
1203	24311601_Intermediate	-8.4

1204	24357240_Accepted	-8.4
1205	22405412_Accepted	-8.4
1206	24780291_Intermediate	-8.4
1207	24268620_Intermediate	-8.4
1208	89449229_Intermediate	-8.4
1209	17410108_Intermediate	-8.4
1210	24356129_Intermediate	-8.4
1211	49820148_Intermediate	-8.4
1212	24345436_Intermediate	-8.4
1213	49724426_Accepted	-8.4
1214	4256987_Intermediate	-8.4
1215	24800100_Intermediate	-8.4
1216	47204706_Intermediate	-8.4
1217	26537660_Accepted	-8.4
1218	7972159_Accepted	-8.4
1219	22407852_Intermediate	-8.4
1220	49641319_Intermediate	-8.4
1221	85256910_Intermediate	-8.4
1222	49821917_Accepted	-8.4
1223	26615613_Intermediate	-8.4
1224	49733789_Intermediate	-8.4
1225	17506829_Intermediate	-8.4
1226	89852199_Intermediate	-8.4
1227	26530481_Accepted	-8.4
1228	4261270_Accepted	-8.4
1229	26649605_Intermediate	-8.4
1230	11110889_Intermediate	-8.4
1231	24786371_Intermediate	-8.4
1232	49672984_Intermediate	-8.4
1233	24798741_Accepted	-8.4
1234	92386620_Intermediate	-8.4
1235	49676817_Accepted	-8.4
1236	17514930_Accepted	-8.4
1237	24289058_Accepted	-8.4
1238	26541161_Accepted	-8.4
1239	49733982_Accepted	-8.4
1240	49680129_Intermediate	-8.4
1241	26729519_Intermediate	-8.4
1242	104233102_Intermediate	-8.4
1243	24794563_Intermediate	-8.4
1244	16952588_Intermediate	-8.4
1245	24337068_Accepted	-8.4
1246	863082_Accepted	-8.4

1247	57261989_Accepted	-8.4
1248	22415987_Accepted	-8.4
1249	49729702_Intermediate	-8.4
1250	24779406_Accepted	-8.4
1251	26649673_Accepted	-8.4
1252	22411358_Intermediate	-8.4
1253	24336476_Intermediate	-8.4
1254	26647104_Intermediate	-8.4
1255	99454455_Intermediate	-8.4
1256	14727730_Accepted	-8.4
1257	22410939_Intermediate	-8.4
1258	24802569_Intermediate	-8.4
1259	14739007_Intermediate	-8.4
1260	99495232_Intermediate	-8.4
1261	14727249_Intermediate	-8.4
1262	49672862_Intermediate	-8.4
1263	57265134_Intermediate	-8.4
1264	49732503_Intermediate	-8.4
1265	4262907_Intermediate	-8.4
1266	24841181_Intermediate	-8.4
1267	24361358_Accepted	-8.4
1268	17482220_Intermediate	-8.4
1269	852912_Accepted	-8.4
1270	24332425_Intermediate	-8.4
1271	85271111_Accepted	-8.4
1272	26648548_Intermediate	-8.4
1273	24831264_Intermediate	-8.4
1274	26619694_Intermediate	-8.4
1275	24832008_Intermediate	-8.4
1276	144096782_Intermediate	-8.4
1277	17460658_Intermediate	-8.4
1278	4256488_Intermediate	-8.4
1279	26661178_Accepted	-8.4
1280	17387793_Accepted	-8.4
1281	17439461_Accepted	-8.4
1282	14740096_Intermediate	-8.4
1283	47200250_Intermediate	-8.4
1284	26648667_Intermediate	-8.4
1285	124896706_Intermediate	-8.4
1286	14727207_Intermediate	-8.4
1287	49817530_Intermediate	-8.4
1288	26617292_Intermediate	-8.4
1289	49677268_Intermediate	-8.4

1290	14743319_Accepted	-8.4
1291	124896823_Intermediate	-8.4
1292	846409_Intermediate	-8.4
1293	99455304_Accepted	-8.4
1294	24812314_Intermediate	-8.4
1295	85199099_Intermediate	-8.4
1296	24779968_Intermediate	-8.4
1297	124391149_Intermediate	-8.4
1298	144204744_Intermediate	-8.4
1299	26637797_Intermediate	-8.4
1300	26527800_Intermediate	-8.4
1301	49672750_Accepted	-8.4
1302	4258912_Intermediate	-8.4
1303	49734985_Intermediate	-8.4
1304	849533_Intermediate	-8.4
1305	17517186_Intermediate	-8.4
1306	7967907_Intermediate	-8.4
1307	26649014_Accepted	-8.4
1308	49724461_Intermediate	-8.4
1309	49678643_Accepted	-8.4
1310	57263155_Intermediate	-8.4
1311	24839435_Intermediate	-8.4
1312	49729883_Intermediate	-8.4
1313	14730335_Intermediate	-8.4
1314	17460783_Intermediate	-8.4
1315	24298314_Intermediate	-8.4
1316	4260358_Intermediate	-8.4
1317	49672464_Intermediate	-8.4
1318	24373857_Intermediate	-8.4
1319	104222918_Intermediate	-8.4
1320	24784031_Intermediate	-8.4
1321	17415181_Intermediate	-8.4
1322	26648437_Intermediate	-8.4
1323	7997613_Intermediate	-8.4
1324	26514224_Intermediate	-8.4
1325	24412550_Intermediate	-8.4
1326	14726337_Accepted	-8.4
1327	99361052_Intermediate	-8.4
1328	26658571_Intermediate	-8.4
1329	49732122_Accepted	-8.4
1330	24836180_Accepted	-8.4
1331	57267605_Intermediate	-8.4
1332	7975468_Intermediate	-8.4

1333	22406875_Intermediate	-8.4
1334	26625269_Accepted	-8.4
1335	852131_Accepted	-8.4
1336	24829967_Accepted	-8.4
1337	843517_Intermediate	-8.4
1338	24802236_Intermediate	-8.4
1339	24285320_Intermediate	-8.4
1340	11534709_Accepted	-8.4
1341	124754159_Intermediate	-8.4
1342	22407955_Intermediate	-8.4
1343	47200971_Accepted	-8.4
1344	26529641_Intermediate	-8.4
1345	24825702_Intermediate	-8.4
1346	26527812_Intermediate	-8.4
1347	26730657_Accepted	-8.4
1348	26647710_Intermediate	-8.4
1349	26730759_Intermediate	-8.4
1350	26649989_Intermediate	-8.4
1351	14725222_Intermediate	-8.4
1352	24344428_Intermediate	-8.4
1353	26535571_Intermediate	-8.4
1354	24800548_Accepted	-8.4
1355	26537926_Accepted	-8.4
1356	4242167_Intermediate	-8.4
1357	47200686_Intermediate	-8.4
1358	17505131_Accepted	-8.4
1359	24282247_Intermediate	-8.4
1360	22416938_Intermediate	-8.4
1361	48409952_Intermediate	-8.4
1362	24268671_Intermediate	-8.4
1363	24825955_Intermediate	-8.4
1364	17471618_Accepted	-8.4
1365	14745708_Intermediate	-8.4
1366	24307606_Intermediate	-8.4
1367	125264911_Intermediate	-8.4
1368	4250445_Intermediate	-8.4
1369	144098385_Intermediate	-8.4
1370	49724867_Intermediate	-8.4
1371	14722065_Intermediate	-8.4
1372	26528497_Intermediate	-8.4
1373	89853240_Accepted	-8.4
1374	24799217_Intermediate	-8.4
1375	7965712_Intermediate	-8.4

1376	92763722_Accepted	-8.4
1377	93577923_Accepted	-8.4
1378	4247254_Intermediate	-8.4
1379	26650522_Intermediate	-8.3
1380	24349205_Intermediate	-8.3
1381	26727499_Intermediate	-8.3
1382	845531_Intermediate	-8.3
1383	26644070_Intermediate	-8.3
1384	49672497_Accepted	-8.3
1385	92117367_Intermediate	-8.3
1386	85198222_Accepted	-8.3
1387	14743934_Intermediate	-8.3
1388	49648905_Intermediate	-8.3
1389	17460161_Intermediate	-8.3
1390	124947980_Accepted	-8.3
1391	24333530_Intermediate	-8.3
1392	24840130_Intermediate	-8.3
1393	26637937_Accepted	-8.3
1394	24821192_Intermediate	-8.3
1395	17458014_Intermediate	-8.3
1396	24310166_Intermediate	-8.3
1397	26651062_Intermediate	-8.3
1398	7968870_Intermediate	-8.3
1399	24347347_Accepted	-8.3
1400	49649300_Intermediate	-8.3
1401	26620742_Intermediate	-8.3
1402	14722718_Intermediate	-8.3
1403	49716187_Intermediate	-8.3
1404	49732544_Accepted	-8.3
1405	49675746_Accepted	-8.3
1406	144090721_Intermediate	-8.3
1407	49674038_Intermediate	-8.3
1408	850226_Intermediate	-8.3
1409	17410114_Intermediate	-8.3
1410	22407071_Intermediate	-8.3
1411	47195519_Intermediate	-8.3
1412	17458027_Intermediate	-8.3
1413	4256530_Intermediate	-8.3
1414	17411342_Accepted	-8.3
1415	4246110_Intermediate	-8.3
1416	17410725_Accepted	-8.3
1417	24834336_Intermediate	-8.3
1418	57260805_Intermediate	-8.3

1419	7969807_Intermediate	-8.3
1420	26628749_Intermediate	-8.3
1421	22416858_Intermediate	-8.3
1422	49718982_Accepted	-8.3
1423	26726983_Accepted	-8.3
1424	26621591_Accepted	-8.3
1425	4248086_Accepted	-8.3
1426	49821342_Accepted	-8.3
1427	24779649_Intermediate	-8.3
1428	26534147_Intermediate	-8.3
1429	17406870_Intermediate	-8.3
1430	26647508_Intermediate	-8.3
1431	49720942_Accepted	-8.3
1432	24294277_Intermediate	-8.3
1433	26647510_Intermediate	-8.3
1434	103050807_Intermediate	-8.3
1435	26535144_Accepted	-8.3
1436	85199644_Intermediate	-8.3
1437	848995_Accepted	-8.3
1438	22412053_Intermediate	-8.3
1439	47193948_Accepted	-8.3
1440	24303739_Intermediate	-8.3
1441	47198740_Intermediate	-8.3
1442	49819722_Intermediate	-8.3
1443	24798366_Intermediate	-8.3
1444	49671408_Intermediate	-8.3
1445	3712285_Intermediate	-8.3
1446	24336997_Intermediate	-8.3
1447	24779569_Intermediate	-8.3
1448	103074742_Intermediate	-8.3
1449	24349015_Accepted	-8.3
1450	49668659_Intermediate	-8.3
1451	17475298_Intermediate	-8.3
1452	14741601_Accepted	-8.3
1453	24818019_Intermediate	-8.3
1454	26537331_Intermediate	-8.3
1455	7968010_Accepted	-8.3
1456	99454304_Intermediate	-8.3
1457	49642625_Intermediate	-8.3
1458	4261238_Intermediate	-8.3
1459	17510353_Intermediate	-8.3
1460	4251065_Intermediate	-8.3
1461	26528270_Intermediate	-8.3

1462	14734096_Intermediate	-8.3
1463	57261489_Intermediate	-8.3
1464	17475181_Accepted	-8.3
1465	26648224_Intermediate	-8.3
1466	24345441_Intermediate	-8.3
1467	24823453_Intermediate	-8.3
1468	47201068_Intermediate	-8.3
1469	26632658_Intermediate	-8.3
1470	859670_Intermediate	-8.3
1471	103075436_Accepted	-8.3
1472	24806916_Intermediate	-8.3
1473	14734982_Intermediate	-8.3
1474	16953520_Intermediate	-8.3
1475	24405598_Intermediate	-8.3
1476	24830956_Intermediate	-8.3
1477	850512_Intermediate	-8.3
1478	26638288_Intermediate	-8.3
1479	24828913_Intermediate	-8.3
1480	49674422_Intermediate	-8.3
1481	49717945_Accepted	-8.3
1482	49827453_Accepted	-8.3
1483	4251095_Intermediate	-8.3
1484	48409945_Intermediate	-8.3
1485	4264845_Accepted	-8.3
1486	14735420_Accepted	-8.3
1487	24394516_Intermediate	-8.3
1488	24800680_Accepted	-8.3
1489	124949808_Intermediate	-8.3
1490	4260300_Intermediate	-8.3
1491	14723543_Intermediate	-8.3
1492	124350391_Accepted	-8.3
1493	26651292_Accepted	-8.3
1494	17481259_Intermediate	-8.3
1495	124947938_Intermediate	-8.3
1496	49822060_Intermediate	-8.3
1497	17458580_Intermediate	-8.3
1498	26539272_Intermediate	-8.3
1499	49820696 _Intermediate	-8.3
1500	22406971_Intermediate	-8.3

Table S1 – List of selected compounds used in the virtual screening.

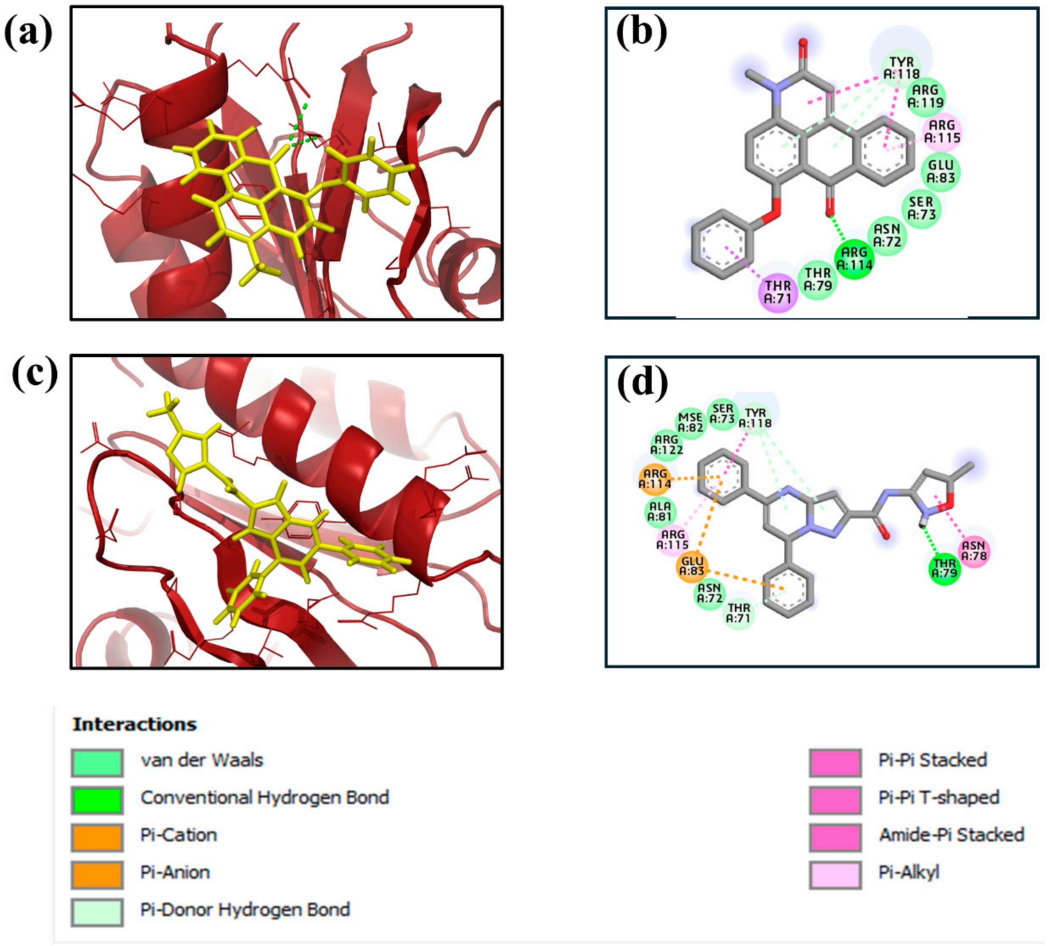

Figure S1 - 3D and 2D structure analysis of viral and host protein (a-b) 4QWO_17444176, (c-d) 4QWO_17450998.

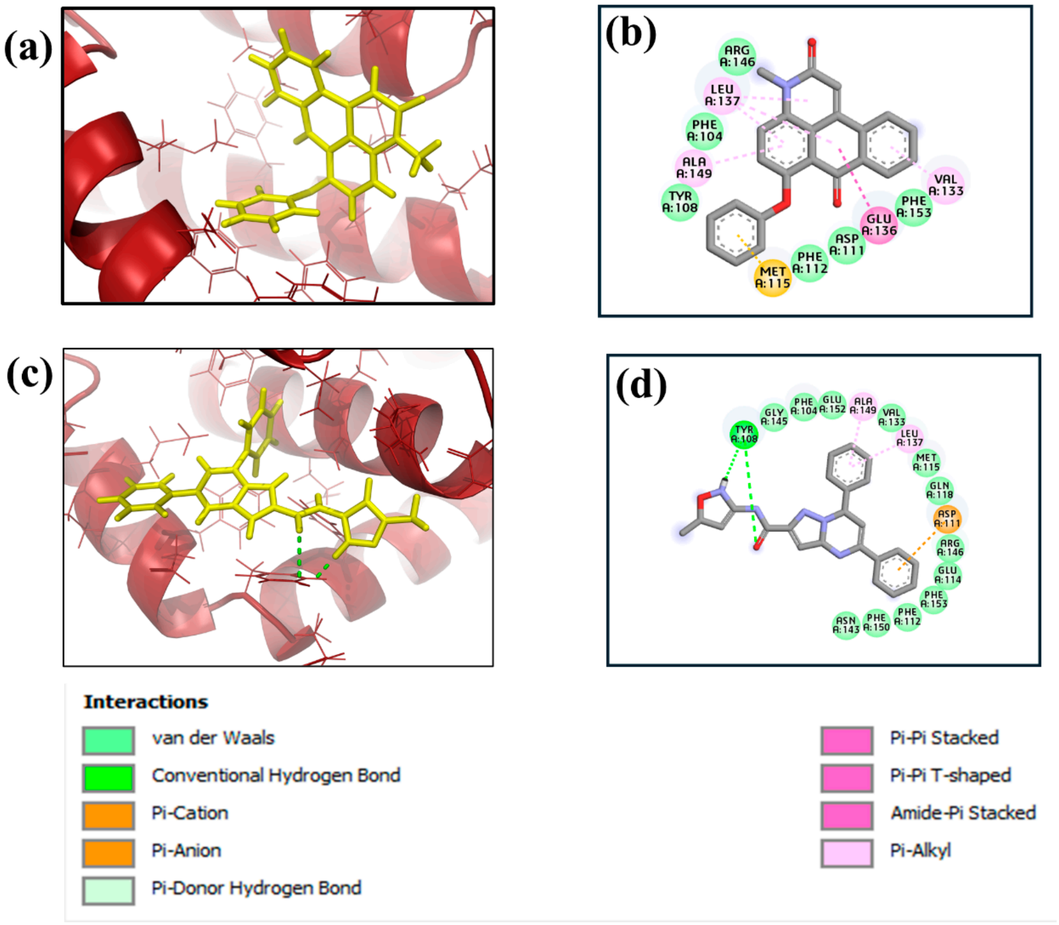

Figure S2 - 3D and 2D structure analysis of viral and host protein (a-b) 8HTS_17444176, (c-d) 8HTS_17450998.

S no.	Complex	H-Bond	Van der Waals	π-π stacking/ π-π cation
1	4QWO_17444176	Arg^{114}	Arg^{119}, Glu^{83}. Ser^{73}, Asn^{72}, Thr^{79}	Tyr^{118}
2	4QWO_17450998	Thr^{79}	Ser^{73}, Arg^{122}, Ala^{81}, Asn^{72}	Asn^{78}, Tyr^{118}

Table S2. Intermolecular analysis of viral and host protein 1- 4QWO_17444176, and 2- 4QWO_17450998

S no.	Complex	H-Bond	Van der Waals	π-π stacking/ π-π cation
1	8HTS_17444176	--	Phe^{153}, Asp^{111}, Phe^{112}, Tyr^{108}, Phe^{104}, Arg^{146}	--
2	8HTS_17450998	Tyr^{108}	Gly^{145}, Phe^{104}, Glu^{152}, Val^{133}, Met^{115}, Gln^{118}, Arg^{146}, Glu^{114}, Phe^{153}, Phe^{112}, Phe^{150}, Asn^{143}	--

Table S3. Intermolecular analysis of viral and host protein 1. 8HTS_17444176, 2. 8HTS_17450998

MM/GBSA components	Control	17450998	24392109	17444176
ΔGBind	-76.80±4.57	-68.92±2.51	-61.55±3.51	-86.19±5.34
ΔGBind Coulomb	-10.06±4.46	-10.42±2.36	-14.95±4.99	-35.19±5.28
ΔGBind Covalent	-0.41±0.25	4.58±1.58	-0.59±1.30	0.88±2.02
ΔGBind Hbond	-0.27±0.26	-0.95±0.47	-1.35±0.57	-4.57±0.75
ΔGBind Lipo	-27.49±1.77	-15.38±0.81	-20.72±0.77	-19.36±1.31
ΔGBind Packing	-4.76±0.48	-6.91±0.26	-3.33±0.68	-1.19±0.13
ΔGBind Solv GB	19.23±2.35	19.73±1.39	27.49±2.58	35.12±5.21
ΔGBind vdW	-53.02±2.09	-59.55±2.38	-48.06±2.58	-61.88±2.76
Ligand Strain Energy	1.43±0.51	3.82±1.50	5.17±0.64	7.30±3.11

Table S4 - MMGBSA analysis of replica 2 for selected compounds in the docked pocket of protein.

MM/GBSA components	Control	17450998	24392109	17444176
ΔGBind	-76.80±4.57	-68.91±2.51	-61.54±3.51	-86.19±5.35
ΔGBind Coulomb	-10.06±4.45	-10.42±2.36	-14.95±4.99	-35.18±5.28
ΔGBind Covalent	-0.41±0.25	4.59±1.57	-0.59±1.30	0.88±2.02
ΔGBind Hbond	-0.26±0.26	-0.95±0.47	-1.35±0.56	-4.57±0.74
ΔGBind Lipo	-27.49±1.77	-15.37±0.81	-20.73±0.77	-19.37±1.31
ΔGBind Packing	-4.76±0.48	-6.91±0.26	-3.33±0.68	-1.18±0.13
ΔGBind Solv GB	19.23±2.35	19.73±1.39	27.49±2.58	35.12±5.21
ΔGBind vdW	-53.01±2.10	-59.56±2.37	-48.06±2.59	-61.88±2.76
Ligand Strain Energy	1.43±0.51	3.83±1.50	5.17±0.65	7.30±3.11

Table S5 - MMGBSA analysis of replica 3 for selected compounds in the docked pocket of protein.

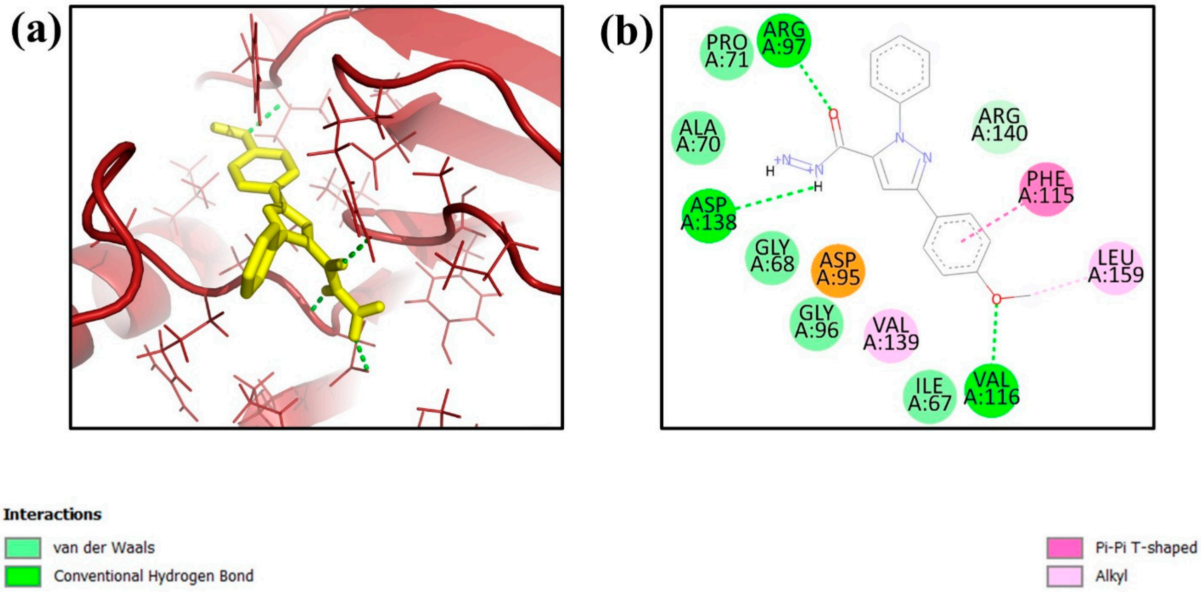

Figure S3 – 2D and 3D molecular interaction plot of negative control compound.

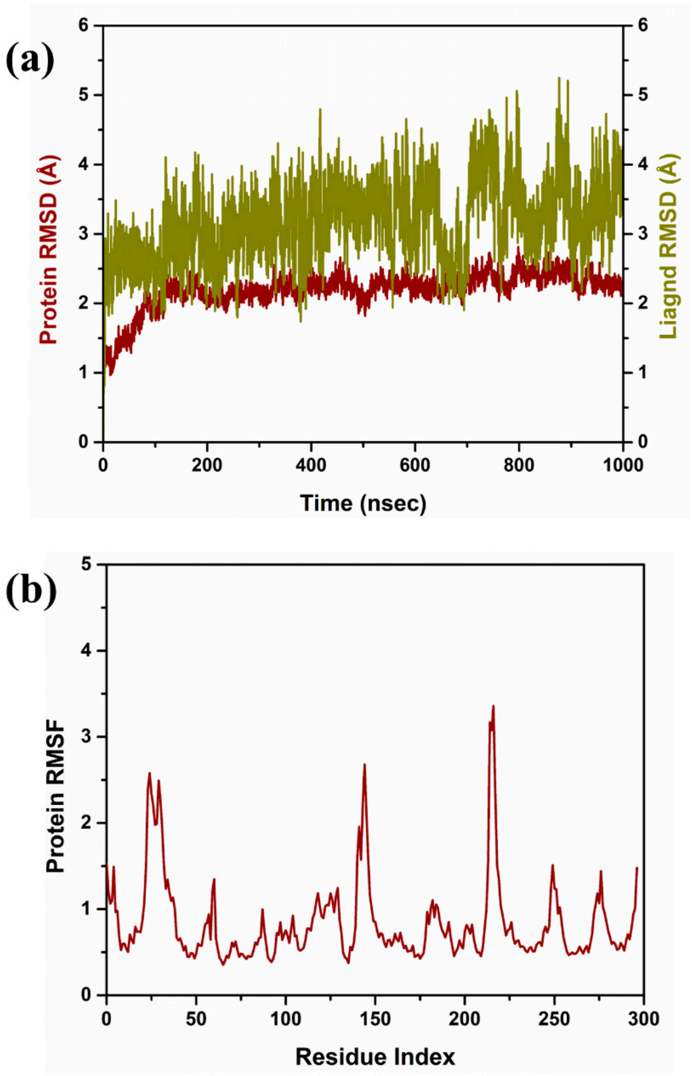

Figure S4 – RMSD AND RMSF analysis of negative control compound

(a)

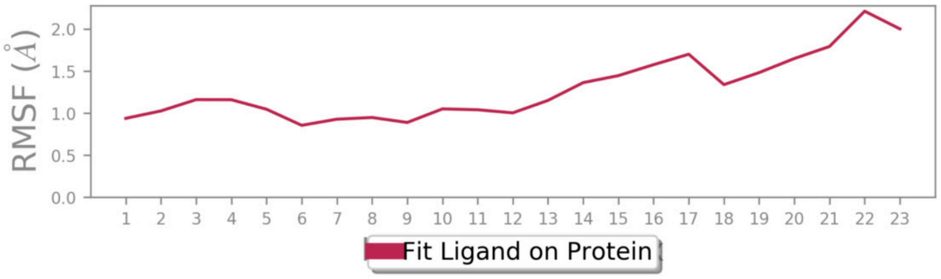

Figure S5 –Ligand RMSF analysis of negative control compound i.e., (a) 22406971.

(a)

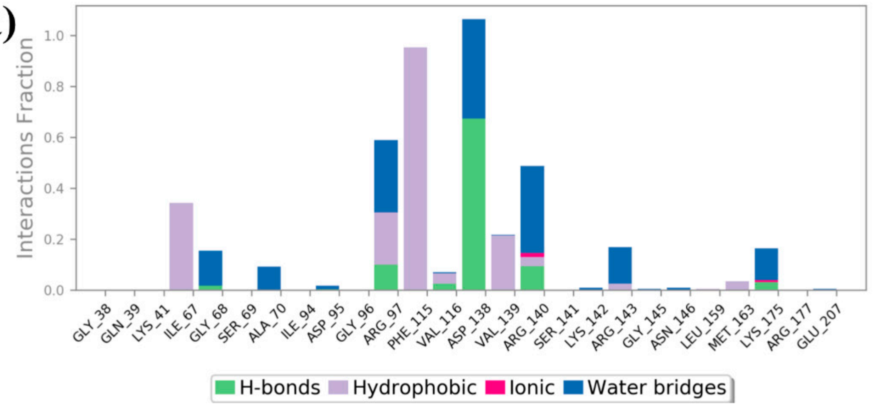

Figure S6 - Protein-ligand profiling of Negative control

(a)

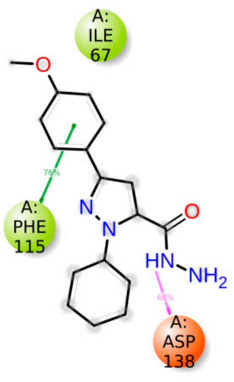

Figure S7 - 2D Protein-ligand interaction analysis of Negative control

(a)

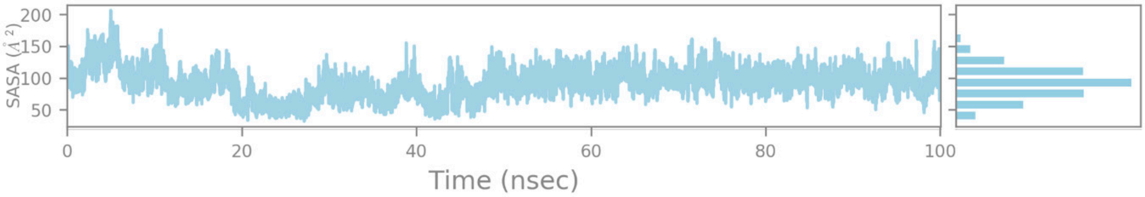

Figure S8 - SASA analysis of Negative control

(a)

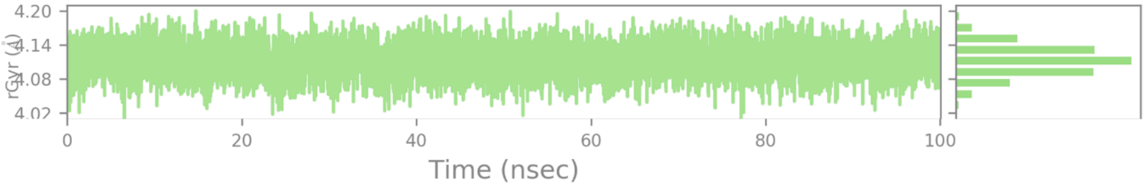

Figure S9 - RG analysis of Negative control

MM/GBSA components	22406971
ΔGBind	-56.33679
ΔGBind Coulomb	-13.65043
ΔGBind Covalent	5.38698
ΔGBind Hbond	-0.35156
ΔGBind Lipo	-21.35693
ΔGBind Packing	-3.00415
ΔGBind Solv GB	21.79099
ΔGBind vdW	-48.4747
Ligand Strain Energy	3.34705

Table S6 - MMGBSA analysis of Negative control
